# Identification of response signatures for tankyrase inhibitor treatment in tumor cell lines

**DOI:** 10.1016/j.isci.2021.102807

**Published:** 2021-07-01

**Authors:** Line Mygland, Shoshy Alam Brinch, Martin Frank Strand, Petter Angell Olsen, Aleksandra Aizenshtadt, Kaja Lund, Nina Therese Solberg, Max Lycke, Tor Espen Thorvaldsen, Sandra Espada, Dorna Misaghian, Christian M. Page, Oleg Agafonov, Ståle Nygård, Nai-Wen Chi, Eva Lin, Jenille Tan, Yihong Yu, Mike Costa, Stefan Krauss, Jo Waaler

**Affiliations:** 1 Department of Immunology and Transfusion Medicine, Oslo University Hospital, P.O. Box 4950 Nydalen, Oslo 0424, Norway; 2 Hybrid Technology Hub - Centre of Excellence, Institute of Basic Medical Sciences, University of Oslo, P.O. Box 1110 Blindern, 0317 Oslo, Norway; 3 School of Health Sciences, Kristiania University College, P.O. Box 1190 Sentrum, 0107 Oslo, Norway; 4 Department of Molecular Cell Biology, Institute for Cancer Research, Oslo University Hospital, Montebello, 0379 Oslo, Norway; 5 Center for Fertility and Health, Norwegian Institute of Public Health, P.O. Box 222 Skøyen, 0213 Oslo, Norway; 6 Oslo Centre for Biostatistics and Epidemiology, Oslo University Hospital, P.O. Box 4950 Nydalen, 0424 Oslo, Norway; 7 Bioinformatics Core Facility, Department of Core Facilities, Institute for Cancer Research, Oslo University Hospital, Ullernchausseen 70, 0379 Oslo, Norway; 8 Department of Informatics, University of Oslo, P.O. box 080 Blindern, 0316 Oslo, Norway; 9 Endocrine Service, VA San Diego Healthcare System, 3350 La Jolla Village Dr., San Diego, CA 92161, USA; 10 Department of Discovery Oncology, Genentech, Inc., 1 DNA Way, South San Francisco, CA 94080, USA

**Keywords:** Proteomics, Cancer, Transcriptomics

## Abstract

Small-molecule tankyrase 1 and tankyrase 2 (TNKS1/2) inhibitors are effective antitumor agents in selected tumor cell lines and mouse models. Here, we characterized the response signatures and the in-depth mechanisms for the antiproliferative effect of tankyrase inhibition (TNKSi). The TNKS1/2-specific inhibitor G007-LK was used to screen 537 human tumor cell lines and a panel of particularly TNKSi-sensitive tumor cell lines was identified. Transcriptome, proteome, and bioinformatic analyses revealed the overall TNKSi-induced response signatures in the selected panel. TNKSi-mediated inhibition of wingless-type mammary tumor virus integration site/β-catenin, yes-associated protein 1 (YAP), and phosphatidylinositol-4,5-bisphosphate 3-kinase/AKT signaling was validated and correlated with lost expression of the key oncogene MYC and impaired cell growth. Moreover, we show that TNKSi induces accumulation of TNKS1/2-containing β*-*catenin degradasomes functioning as core complexes interacting with YAP and angiomotin proteins during attenuation of YAP signaling. These findings provide a contextual and mechanistic framework for using TNKSi in anticancer treatment that warrants further comprehensive preclinical and clinical evaluations.

## Highlights

• TNKSi-responding tumor cell lines were identified
• TNKSi targets WNT/β-catenin, YAP, and PI3K/AKT signaling
• Reduced MYC expression leads to impaired tumor cell growth

## Graphical abstract

**Figure:**
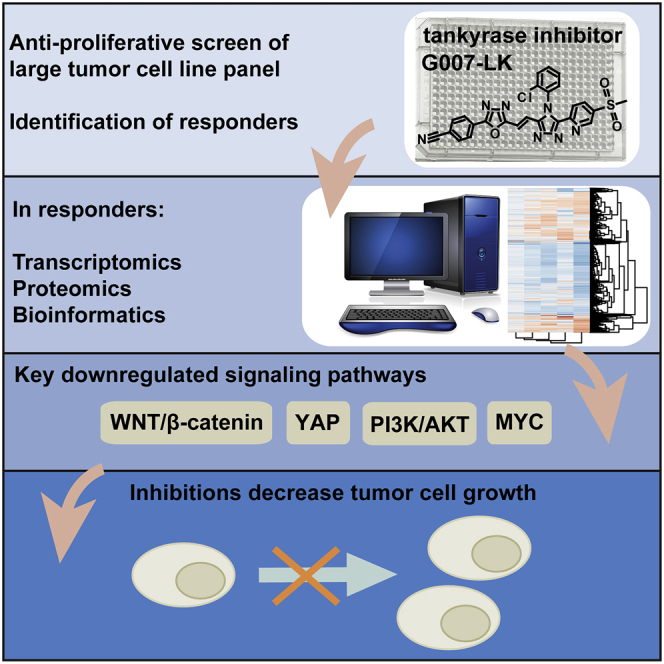

## Introduction

Anticancer treatment, using small-molecule tankyrase 1 and tankyrase 2 (TNKS1/2) inhibitors, shows *in vivo* efficacy against colorectal cancer (Lau et al., 2013; Waaler et al., 2012) and osteosarcoma (Martins-Neves et al., 2018) in mouse xenograft models. The therapeutic effect can be enhanced and broadened by combining tankyrase inhibition (TNKSi) with inhibitors of phosphatidylinositol-4,5-bisphosphate 3-kinase (PI3K), epidermal growth factor receptor, or mitogen-activated protein kinase against colorectal cancer xenografts (Schoumacher et al., 2014; Solberg et al., 2018). Recently, combining TNKSi with antibody-based inhibition of programmed cell death 1 has shown effect in syngeneic melanoma mouse models (Waaler et al., 2020b).

TNKS1/2 are members of the poly (ADP-ribose) polymerase (PARP) family of enzymes that control protein turnover and activities by catalyzing the post-translational modification poly-ADP-ribosylation (Haikarainen et al., 2014; Smith et al., 1998). The poly-ADP-ribose signal is subsequently recognized by the E3 ubiquitin ligase ring finger protein 146 leading to polyubiquitination of the target protein and subsequent proteasomal degradation (Callow et al., 2011; Haikarainen et al., 2014; Nie et al., 2020; Zhang et al., 2011). Independent of the catalytic activity, TNKS1/2 also provide structure-based scaffolding functions (Mariotti et al., 2016; Pollock et al., 2019; Seimiya and Smith, 2002).

Multiple potent small-molecules have been developed to target the catalytic site of TNKS1/2 (Bregman et al., 2013; Huang et al., 2009; Johannes et al., 2015; Mizutani et al., 2018; Shultz et al., 2013; Voronkov et al., 2013; Waaler et al., 2020a). Among these, the triazole-based series including JW74 (Waaler et al., 2011), G007-LK (Voronkov et al., 2013), OD336 (compound 16) (Anumala et al., 2017), and OM-1700 (compound 13) (Waaler et al., 2020a) target the adenosine binding pocket of the TNKS1/2 catalytic domain with high selectivity, whereby G007-LK shows a favorable pharmacokinetic profile in mice (Voronkov et al., 2013). In contrast, agents like XAV939, that bind to the nicotinamide binding pocket of the catalytic domain, are less selective in that they also inhibit additional PARP family members (Haikarainen et al., 2014; Huang et al., 2009).

Despite the progress in structural development, no TNKS1/2 inhibitor has yet entered clinical use for any application (Ferri et al., 2017). Clinical tankyrase inhibitor development has so far been hampered by concerns over intestinal toxicity and other on-target or signaling-pathway-specific side effects (Fujita et al., 2018; Lau et al., 2013; Zhong et al., 2016a, 2016b). Although current preclinical stage tankyrase-specific inhibitors, including G007-LK, do not display the chemical properties required for approval for human testing (Voronkov et al., 2013), research to develop additional TNKS1/2 inhibitors for clinical use is actively pursued (Ferri et al., 2017; Waaler et al., 2020a).

TNKS1/2 can orchestrate the activities of several biological mechanisms including proliferation, differentiation, energy metabolism, vesicle transport, telomere homeostasis, and mitotic spindle formation through a multitude of direct poly-ADP-ribosylation targets (Haikarainen et al., 2014; Kim, 2018; Wang et al., 2020; Zimmerlin and Zambidis, 2020). Importantly, TNKSi has been reported to inhibit key cancer-promoting signaling pathways (Sanchez-Vega et al., 2018), such as the wingless-type mammary tumor virus integration site (WNT)/β*-*catenin pathway (Huang et al., 2009), the yes-associated protein 1 (YAP) pathway (Wang et al., 2015), the PI3K/AKT serine/threonine kinase 1 (AKT) pathway (Li et al., 2015), and the notch receptor (NOTCH) pathway (Bhardwaj et al., 2017). In addition, TNKSi-induced AMP-activated protein kinase (AMPK) activation, followed by suppression of tumorigenesis, has been described (Li et al., 2019).

In WNT/β*-*catenin signaling, AXIN1 and AXIN2 proteins control the formation of the β-catenin destruction complex, also termed as β-catenin degradasome, which also contains adenomatous polyposis coli (APC) and glycogen synthase kinase 3 beta (GSK3β) (Lee et al., 2003; Thorvaldsen et al., 2015). TNKS1/2 poly(ADP-ribosyl)ate AXIN1 and AXIN2 proteins leading to their degradation and subsequent activation of WNT/β*-*catenin signaling (Huang et al., 2009). Hence, TNKSi results in stabilization of AXIN proteins, β*-*catenin degradasome accumulation and blockade of WNT/β*-*catenin signaling (Huang et al., 2009; Thorvaldsen et al., 2015). In the Hippo signaling pathway, TNKS1/2 similarly poly-ADP-ribosylate and induce degradation of angiomotin (AMOT), angiomotin-like 1 (AMOTL1), and angiomotin-like 2 (AMOTL2) proteins (Wang et al., 2015). Consequently, TNKSi stabilizes AMOT proteins that in turn change the subcellular location of the transcription cofactors YAP and WW domain containing transcription regulator 1 (WWTR1, also known as TAZ), resulting in decreased YAP signaling activity (Troilo et al., 2016; Wang et al., 2015). Notably, WNT/β*-*catenin and YAP signaling may interface in the β-catenin degradasome: In WNT/β*-*catenin signaling-inactive cells, YAP and TAZ can accumulate in the β-catenin degradasome, while in WNT/β*-*catenin signaling-active cells, YAP and TAZ can dislocate from the β-catenin degradasome leading to their nuclear accumulation and YAP signaling activation (Azzolin et al., 2014). In the PI3K/AKT signaling pathway, TNKS1/2 were reported to target and induce turnover of phosphatase and tensin homolog (PTEN), a phosphatase that opposes PI3K/AKT signaling antagonist (Li et al., 2015). As a consequence, AKT could be inactivated by TNKSi-mediated stabilization of PTEN (Li et al., 2015).

The master transcriptional regulator MYC proto-oncogene (MYC) is deregulated in >50% of human cancers, in line with a central function in controlling a multitude of oncogenic processes including differentiation, proliferation, and apoptosis (Chen et al., 2018). WNT/β-catenin, YAP, and PI3K/AKT signaling pathways are all promoters of *MYC* transcription in cancer cells (He et al., 1998; Huh et al., 2019; Kress et al., 2015; Neto-Silva et al., 2010).

The vast majority of studies on the antitumor effect of TNKSi focus on the impact on individual signaling pathways rather than examining the overall downstream biological effects of TNKSi. Here, we used the TNKS1/2-selective inhibitor G007-LK to screen 537 tumor cell lines for an antiproliferative effect and identified a subset highly TNKSi-sensitive cell lines originating from the colon, kidney, ovary, and lung. In this subset, functional and molecular analyses revealed that TNKSi can context-dependently antagonize the oncogenic signaling pathways WNT/β-catenin, YAP, and PI3K/AKT leading to an impediment of MYC-driven cell growth.

## Results

### Proliferation screen identifies human tumor cell lines susceptible to growth inhibition by the selective tankyrase inhibitor G007-LK

**Figure 1:**
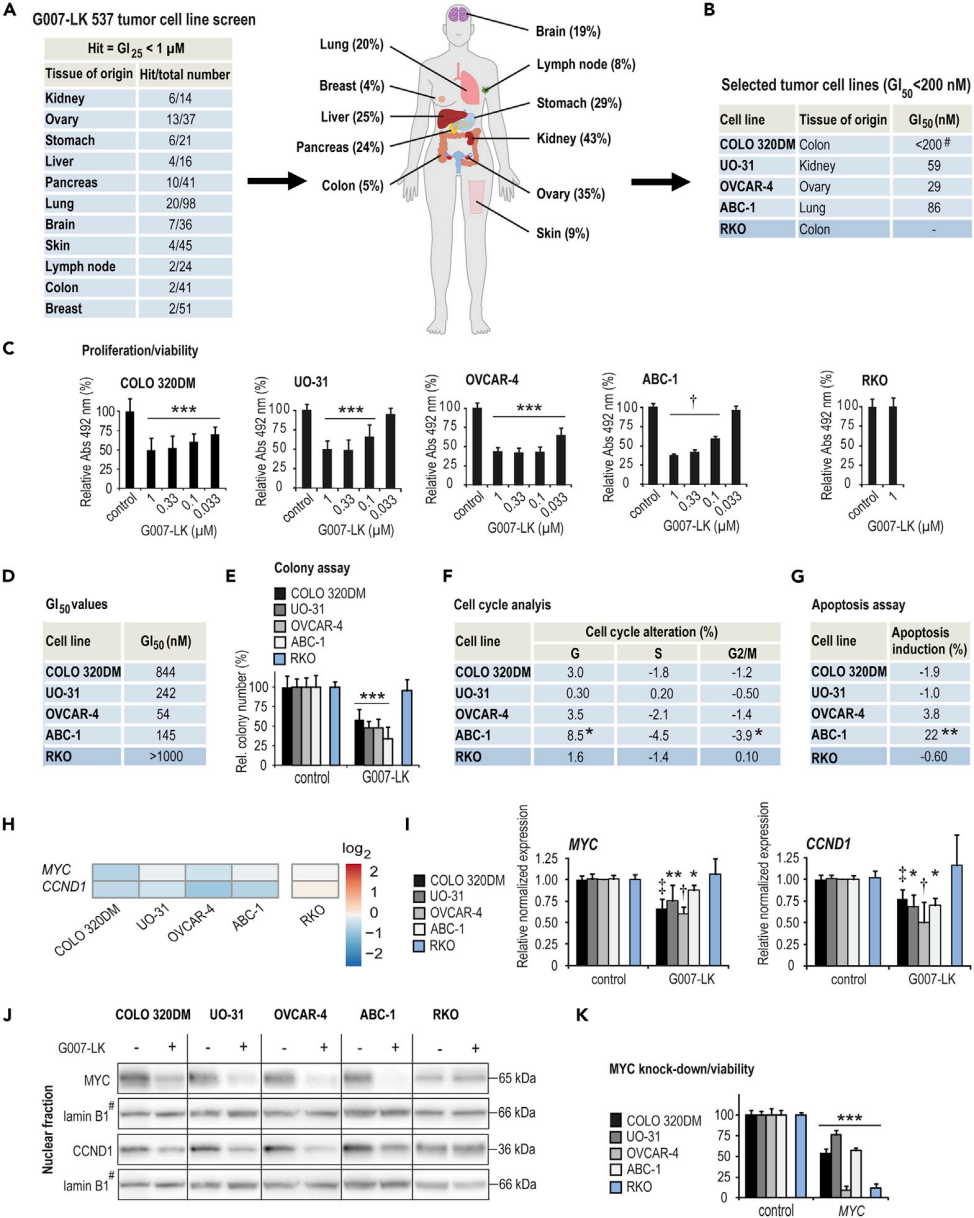
Proliferation screen identifies human tumor cell lines susceptible to growth inhibition by the selective tankyrase inhibitor G007-LK. (A) Proliferation/viability screen: NCI-60 and Genentech tumor cell line panels were treated with G007-LK for 48 and 72 hr respectively. The number (table) and percentage (graph) of GI _25_ value hits (25% cell growth inhibition at a concentration <1 μM G007-LK) versus the total number of tumor cell lines established from various tissues (only tissues represented by ≥ 14 cell lines are shown). (B) Tumor cell lines were selected for further analysis (GI _50_ values [50% cell growth inhibition] at < 200 nM G007-LK, light blue) including COLO 320DM cells (Lau et al., 2013) and in comparison with RKO control cells (blue). (C) Re-screening using endpoint MTS proliferation assay (Abs_492_) for the indicated concentrations of G007-LK for 4–8 days relative to control (100%, 0.01% DMSO) and experiment time 0 values (t_0_, set to 0%). One-way ANOVA tests (Holm-Sidak method versus control) are indicated by ∗∗∗ (p < 0.001) and one-way ANOVA on ranks tests (Dunn's method versus control) are indicated by † (p < 0.05). Mean values ±SD for one representative experiment of at least two repeated assays, each with six replicates, are shown. (D) Endpoint MTS proliferation assay GI 50-values. (E) Relative colony numbers (%) upon 7–11 days of treatment with G007-LK (1 μM) compared to DMSO (0.01%). Mean values ±SD for combined data from a minimum of three independent experiments with three replicates each are shown. For **E–G**, **I,** and **K**, two-tailed t-tests are indicated by ∗∗∗ (p < 0.001), ∗∗ (p < 0.01), and ∗ (p < 0.05) while Mann-Whitney rank-sum tests are indicated by ‡ (p < 0.01) and † (p < 0.05). (F) Cell cycle alteration relative to control (%). G = gap_1_ phase, S = synthesis phase, G2/M = gap_2_/mitosis phase. Mean values from combined data consisting of a minimum of four independent experiments are shown. For **F** and **G**, upon 72-h treatment with G007-LK (1 μM) compared control (set to 0%, 0.01% DMSO). (G) Induction of apoptosis relative to control. Mean values from combined data consisting of a minimum of three independent experiments are shown. (H) RNA sequencing analysis of the cell cycle-controlling genes *MYC* and *CCND1* (log_2_, n = 2). For **H–J**, after 24 h of treatment with G007-LK (1 μM) or controls (0.01% DMSO). (I) Real-time RT-qPCR analyses of *MYC* and *CCND1*. Mean values ±SD for combined data from minimum two independent experiments and measurements with three replicates each are shown. (J) Immunoblots of nuclear MYC and CCND1 using lamin B1 documenting protein loading, while # indicates that the same lamin B1 immunoblot is used as loading control for both MYC and CCND1. Representative data from two or more independent experiments are shown. (K) Endpoint MTS proliferation assay, (Abs_492_) relative to control (100%) and experiment time 0 values (t_0_, set to 0%), 5 days after transfection with siRNA against *MYC* and *EGFP* (control). Mean values ±SD for one experiment representative of a minimum of two independent assays are shown. See also Table S1 and Figure S1.

Previous reports have shown that TNKSi can block proliferation and reduce viability in a limited subset of cancer cell lines *in vitro* (Kim, 2018; Nusse and Clevers, 2017). However, little is known regarding the antiproliferative effect of TNKSi against a vast number of cancer types, let alone the subsets within each cancer type. To evaluate TNKSi-mediated inhibition of cell growth, the TNKS1/2-specific inhibitor G007-LK was screened against a panel of 537 human tumor cell lines, including the NCI-60 tumor cell line panel. These human tumor cell lines originated from 29 different tissues bearing various primary diagnoses. The concentrations of G007-LK treatment that inhibited cell growth by 25% or 50% (GI_25_ and GI_50_ values) were determined. Out of the 537 tested tumor cell lines, 87 (16%) displayed GI _25_ values < 1 μM G007-LK. These included >20% of the cancer cell lines originating from the kidney, ovary, stomach, liver, pancreas and lung (Figure 1A and Table S1). The screening results suggest that TNKSi obstructed the growth of a broad range of cancer types *in vitro*.

The proliferation screen identified three cell lines that were particularly susceptible to the growth-inhibitory effect of G007-LK (TNKSi-sensitive) with GI _50_ values < 200 nM. These three cell lines, UO-31 (renal cancer), OVCAR-4 (ovarian cancer), and ABC-1 (non-small-cell lung cancer), along with the previously identified benchmark TNKSi-sensitive cell line COLO 320DM (Lau et al., 2013) (colon cancer), were submitted to subsequent analyses to identify the mechanisms that render them particularly sensitive to TNKSi (Figure 1B and Table S1). The TNKSi-insensitive colon cancer cell line RKO was included as a negative control (Mizutani et al., 2018; Solberg et al., 2018; Tanaka et al., 2017). To verify the screening data, the panel was subsequently rescreened. In the retested cell lines, G007-LK significantly decreased cell growth, as measured by colorimetric MTS viability (GI_50_ values: 54–844 nM) and colony assays (42–66% reduction), in all TNKSi-sensitive cell lines, while control RKO cells remained unaffected by the treatment (Figures 1C, 1D, 1E, and S1A).

Next, cell cycle and apoptosis analyses were performed to further investigate TNKSi-induced cell growth inhibition. In the panel of TNKSi-sensitive cell lines, only ABC-1 cells exhibited significant G_1_ cell cycle arrest and apoptosis upon G007-LK treatment (Figures 1F, 1G, S1B, and S1C). By contrast, RNA sequencing and real-time qRT-PCR analyses revealed significantly reduced transcripts of the key cell-cycle-promoting genes *MYC* and *cyclin D1* (*CCND1*), as well as MYC and cyclin D1 protein in all selected TNKSi-sensitive cell lines after G007-LK treatment, but not in RKO control cells, suggesting that TNKSi is unable to block MYC expression in RKO cells (Figures 1H–1J and S1D). Finally, to examine whether decreased MYC expression can impair cell growth, the selected cell panel was transfected with siRNA against *MYC*. Knockdown of *MYC*, to recapitulate the G007-LK-mediated reduction of MYC protein, resulted in a significant inhibition of cell growth in all cell lines, also in RKO control cells (Figure 5K and S1E). The results indicate that all tested cell lines depend on expression of MYC for sustained cell proliferation.

In conclusion, the results suggest that TNKSi decreases MYC and cyclin D1 expression leading to induction of cytotoxic G_1_ cell cycle arrest and apoptosis in ABC-1 cells, while overall slower cell cycle progression is the primary cause of the cytostatic cell growth inhibition observed in COLO 320DM, UO-31, and OVCAR-4 cells.

### Gene expression analysis reveals that TNKSi attenuates MYC and WNT/β-catenin, YAP, and PI3K/AKT signaling pathways

The effect of TNKSi against tumor cell proliferation may depend on the tumor type, mutation load, the context in which the tumor cells are grown, and the intrinsic activities of various cellular pathways. To map changes in gene expression, proteome, and cell signaling pathways and to correlate them with oncogenic mutations, the selected cell line panel was exposed to G007-LK treatment followed by RNA sequencing, bioinformatic analyses, and proteomics analyses.

**Figure 2:**
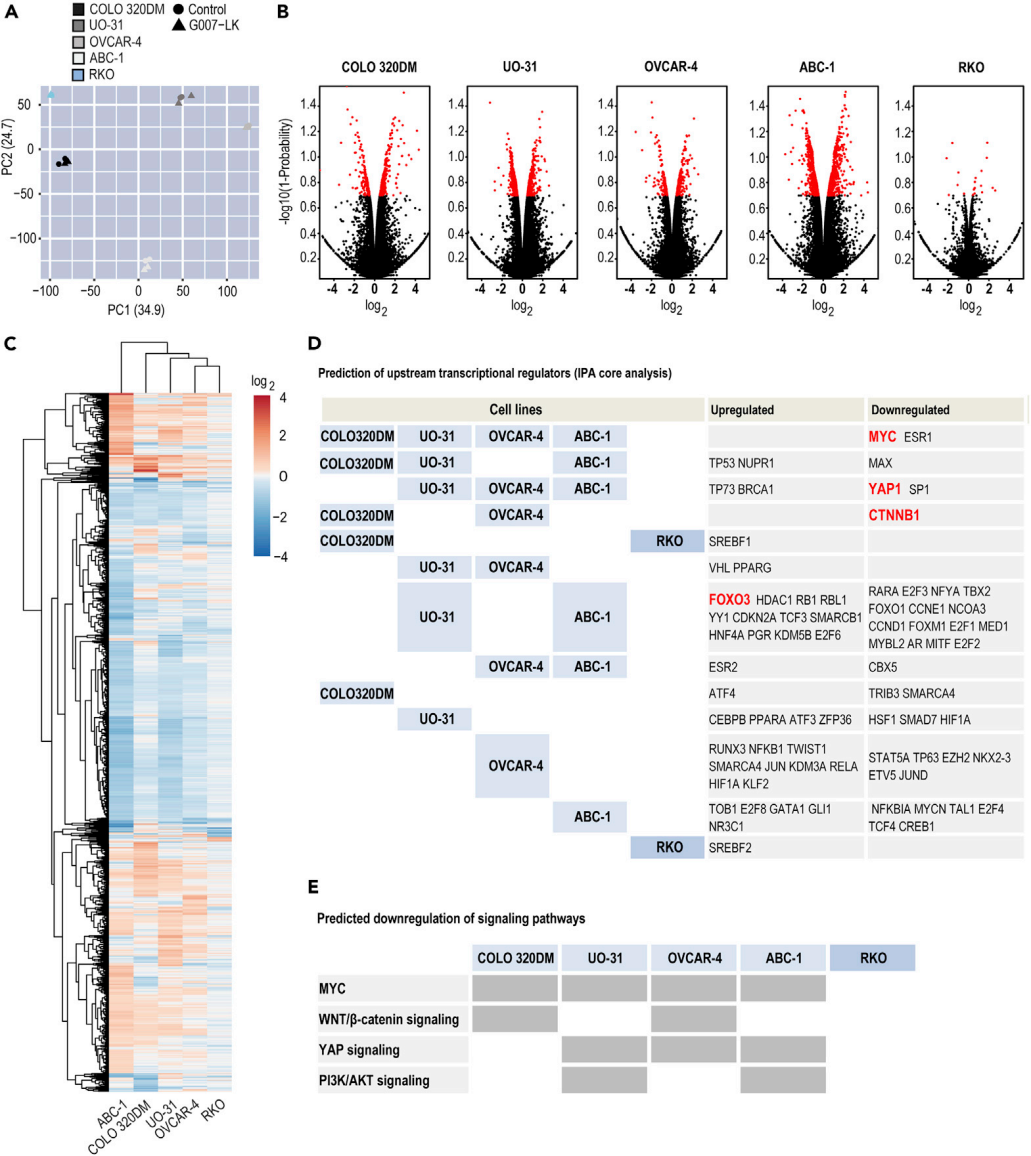
Gene expression analysis reveals that TNKSi attenuates MYC and WNT/β-catenin, YAP and PI3K/AKT signaling pathways (A) Principal component (PC) analysis plot showing gene expression diversity. For **A**–**C**, mRNA sequencing data for selected human tumor cell line panel treated for 24 h with G007-LK (1 μM, n = 2) compared to control (0.01% DMSO, n = 2). (B) Volcano plots showing the effect of G007-LK treatment on gene expression. Transformed NOISeq probability values (-log10[1-Probability]) are plotted against log_2_ fold change. Genes with probability values >0.8 are highlighted in red. (C) Heatmap of log_2_ fold change of genes differentially expressed in one or more cell lines. (D) Prediction of upstream signaling pathway protein regulators (IPA core analysis) based on differentially expressed genes identified in **B** and categorized using Venn analysis. Threshold: Probability values >0.8, p value of overlap <1 × 10^−8^ and absolute activation *Z* score > 0.5 or < −0.5. Key signaling pathway proteins identified are highlighted in red. (E) Prediction of predominantly downregulated signaling pathways. See also Tables S2–S4 and Figure S2.

First, a mutation analysis of the RNA sequencing data set was performed by matching mutations identified in the selected cell lines against a set of previously defined driver oncogenes (Bailey et al., 2018). However, apart from the relative abundant mutations in TNKSi-insensitive RKO cells, no telltale mutational patterns unifying the 5 cell lines were identified (Table S2). Moreover, when comparing the RNA sequencing data, a principal component analysis revealed highly different pretreatment and post-treatment transcriptional profiles (Figure 2A).

Next, to categorize TNKSi-induced transcriptional signatures, a bioinformatics analysis identified 369–1547 statistically significant differently expressed genes (DEGs) in TNKSi-sensitive cells, while only 29 DEGs were found in RKO control cells (Figures 2B and S2A and Table S3). Again, no robust clustering of the DEG profiles leading to a clear subclassification of the cell lines was observed (Figures 2C).

An Ingenuity Pathway core Analysis (IPA) was therefore applied, based on the DEG transcript profiles uncovered by RNA sequencing, to identify statistically significant key upstream transcriptional regulating proteins. In all four selected TNKSi-sensitive cell lines, estrogen receptor 1 (ESR1) and MYC were predicted to be downregulated upstream regulators by TNKSi (Figures 2D [see top row], S2B and Table S3). For three out of the four TNKSi-sensitive cell lines, activities of four proteins were predicted to be upregulated upstream regulators after G007-LK treatment: Tumor protein p53 (TP53), nuclear protein 1, transcriptional regulator (NUPR1), tumor protein p73 (TP73), and BRCA1 DNA repair associated. Three proteins were predicted to be downregulated upstream regulators: MYC-associated factor X (MAX), YAP1, and Sp1 transcription factor (SP1) (Figures 2D and S2B and Table S3). Several of these identified upstream regulator proteins are known to control apoptosis and cell cycle, such as TP53 (Hafner et al., 2019), TP73 (Rodriguez et al., 2018), and MYC (Chen et al., 2018), while MAX is associated with MYC in the MYC-MAX complex (Cascon and Robledo, 2012).

Transcription of *MYC* can be regulated by several signaling pathways that contain tankyrase target proteins including WNT/β-catenin, YAP, and PI3K/AKT signaling (He et al., 1998; Huh et al., 2019; Kress et al., 2015; Neto-Silva et al., 2010). Within these pathways, CTNNB1 (β-catenin) was predicted to be a downregulated upstream regulator in COLO 320DM and OVCAR-4 cells upon G007-LK treatment (Figures 2D, 2E, and S2B and Table S3). Several of the upstream regulator proteins, predicted in the IPA core analysis, are associated with WNT/β-catenin signaling activity: SP1 is regulated by the β-catenin destruction complex (Mir et al., 2018), ESR1 is involved in cross talk with WNT/β-catenin signaling, while forkhead box O3 (FOXO3) can bind and interact with β-catenin (Kouzmenko et al., 2004; Valenta et al., 2012). YAP1 was predicted to be a downregulated upstream regulator in UO-31, OVCAR-4, and ABC-1 cells after exposure to G007-LK (Figures 2D and S2B and Table S3), while NUPR1 transcription is controlled by YAP signaling (Jia et al., 2016). FOXO3, a central effector of PI3K/AKT signaling (Stefanetti et al., 2018), was predicted to be an upregulated upstream regulator in UO-31 and ABC-1 cells (Figures 2D, 2E, and S2B and Table S3). In a previous report, decreased NOTCH signaling was observed in a proteome analysis of TNKS1/2 knockout HEK293 cells (Bhardwaj et al., 2017). However, predictions of NOTCH1 activity were outside the threshold level used in the IPA core analysis for all cell lines (Table S3), and no distinct downregulation of NOTCH signaling target genes was observed in any of the cell lines (Figure S2C).

Finally, an SILAC-based proteome analysis identified 590–847 statistically significant differently expressed proteins in the selected TNKSi-sensitive cells, while 501 proteins were found in RKO cells (Figure S2D and Table S4). No robust clustering classifying the cell line's protein expression profiles was observed (Figure S2E). However, among upregulated proteins, the energy metabolism-regulating proteins transketolase, NADH:ubiquinone oxidoreductase subunit A8, and hydroxyacyl-CoA dehydrogenase trifunctional multienzyme complex subunit beta were identified in a minimum of four of the cell lines after G007-LK treatment (Table S4). Previous reports have shown TNKSi-mediated regulation of energy metabolism in mouse models (Wang et al., 2020; Zhong et al., 2016a, 2016b).

In conclusion, the analysis of transcriptional responses to G007-LK exposure indicates a repertoire of rather diverse regulation of signaling pathways in TNKSi- sensitive tumor cell lines. Nevertheless, TNKSi predominantly leads to cell type-dependent and primary inhibition of the WNT/β-catenin, YAP, and PI3K/AKT signaling pathways and, subsequently, counteraction of MYC-driven cell cycle progression and tumor cell growth (Figure 2E). Hence, the effect of G007-LK against these three signaling pathways was further investigated in the selected cell line panel.

### G007-LK inhibits WNT/β-catenin signaling in a subset of tumor cell lines that are dependent on β-catenin for sustained cell growth

**Figure 3:**
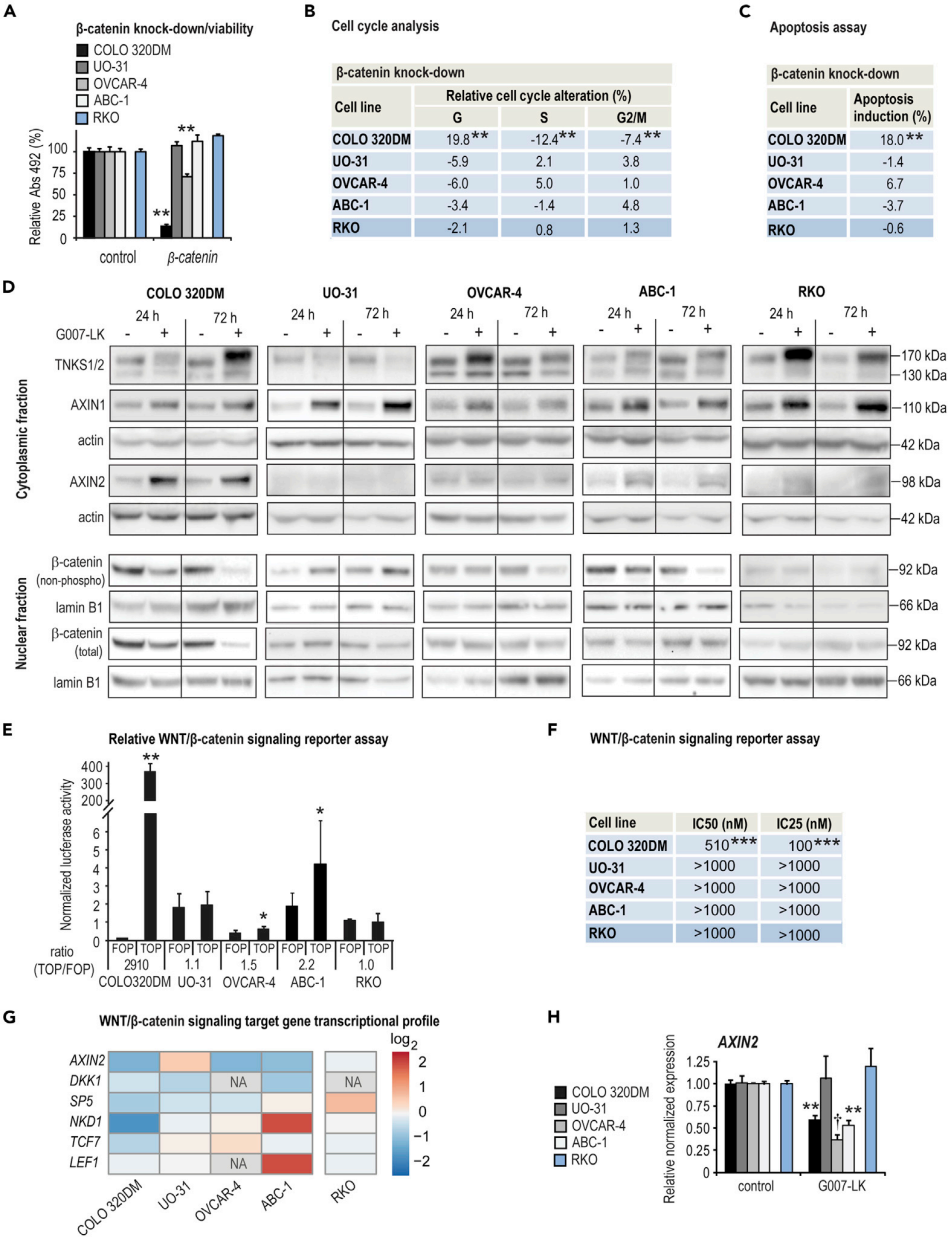
G007-LK inhibits WNT/β-catenin signaling in a subset of tumor cell lines that are dependent on β-catenin for sustained cell growth (A) Endpoint MTS proliferation assay (Abs_492_) relative to control (100%) and experiment time 0 values (t_0_, set to 0%). Mean values ±SD for one experiment representative of a minimum of two independent assays are shown. For **A**–**C**, 5–8 days after transfection with siRNA against *CTNNB1* and *EGFP* (control). For **A**–**C**, **E**, **F,** and **H**, two-tailed t-tests as indicated by ∗∗∗ (p < 0.01), ∗∗ (p < 0.01) and ∗ (p < 0.05) while Mann-Whitney rank-sum test is indicated by † (p < 0.05). (B) Cell cycle alteration (%) relative to control (set to 0%). Mean values from combined data consisting of a minimum of four independent experiments are shown. (C) Induction of apoptosis (%) relative to control (set to 0%). Mean values from combined data consisting of a minimum of three independent experiments are shown. (D) Immunoblots of cytoplasmic TNKS1/2, AXIN1 and AXIN2 (upper panels), and nuclear active form of β-catenin (non-phospho[Ser33/37/Thr41]) and total β-catenin (lower panels), after 24 or 72 hr treatment with G007-LK (1 μM) compared to controls (0.01% DMSO). Actin (cytoplasmic) and lamin B1 (nuclear) document protein loading. Representative data from two or more independent experiments are shown. (E) Luciferase-based reporter assay for comparing baseline WNT/β-catenin signaling activity. The cells were transiently co-transfected with either a superTOPflash (vector with 7 X TCF promoter binding sites driving the firefly luciferase) or a FOPflash (control vector with mutated TCF binding sites) along with *Renilla* luciferase (for normalization). All samples are relative to normalized superTOPflash signal for RKO cells (= 1). Mean values ±SD for combined data from 2–4 independent experiments with three replicates each are shown. Statistically significant differences between SuperTOPflash and FOPflash activities (TOP/FOP ratio) are indicated. (F) IC_50_ and IC_25_ values (nM) for luciferase-based WNT/β-catenin signaling reporter assay (stably transfected with SuperTOPflash and *Renilla* luciferase) upon exposure to different concentrations of G007-LK for 72 h compared to control (0.01% DMSO). (G) WNT/β-catenin signaling target gene transcription (RNA sequencing, log_2_) after 24-h treatment with G007-LK (1 μM) compared to controls (0.01% DMSO) (n = 2). NA = not available, no RNA detection. (H) Real-time RT-qPCR analysis of *AXIN2* upon 24 hr G007-LK treatment (1 μM) relative to control (0.01% DMSO). Mean values ±SD for combined data from two independent experiments with three replicates each are shown. See also Figures 4, S3, and S4.

Following G007-LK exposure, β-catenin was predicted by the IPA core analysis to be a downregulated upstream regulator in COLO 320DM and OVCAR-4 cells, indicating reduced WNT/β-catenin signaling (Figure 2). To evaluate if reduced WNT/β-catenin signaling controls cell growth, the cell line panel was subjected to siRNA-mediated knock down of *CTNNB1* to recapitulate G007-LK-mediated β-catenin reduction (Figure S3A). Cell growth was significantly inhibited in only COLO 320DM and OVCAR-4 cells (Figures 3A and S3B), while significant G_1_ cell cycle arrest and induction of apoptosis was observed only in COLO 320DM cells (Figures 3B, 3C, S3C, and S3D).

Western blot analysis was applied to the selected cell line panel to explore the effect of G007-LK treatment on its intended targets TNKS1/2 and also WNT/β-catenin signaling. G007-LK treatment induced, as previously shown (Lau et al., 2013), either a stabilization or a destabilization of TNKS1/2 in all cell lines (Figure 3D). AXIN1 was stabilized in all cell lines, while stabilization of AXIN2 and destabilization of the inactive and phosphorylated form of GSK3β was detected in only COLO 320DM and ABC-1 cells (Figures 3D and S4A). β-catenin was reduced in COLO 320DM cells after 24-h exposure to G007-LK, while a moderate reduction was seen in OVCAR-4 and ABC-1 cells after 72-h treatment (Figures 3D and S4A).

To assess endogenous WNT/β-catenin signaling pathway activities, the cell line panel was transiently cotransfected with a vector containing WNT/β-catenin signaling-responsive promoter driving firefly luciferase expression (superTOPflash), or control vector (FOPflash), along with *Renilla* luciferase for normalization. COLO 320DM cells (APC^mutated^) demonstrated high luciferase activity compared to RKO cells (APC^wild-type^), indicating high endogenous WNT/β-catenin signaling activity (Figure 3E). OVCAR-4 and ABC-1 cells showed moderate but significant increases in superTOPflash signal when compared to the FOPflash signal, suggesting rather low endogenous WNT/β-catenin signaling activities (Figure 3E). In stable superTOPflash and *Renilla* luciferase transfectants, a decrease in WNT/β-catenin signaling activity was only seen in COLO 320DM cells exposed to various doses of G007-LK (Figures 3F and S4C). Although transcription of *AXIN2*, a cell type-universal and negative-feedback-controlling target gene, was significantly reduced in COLO 320DM, OVCAR-4, and ABC-1 cells, RNA sequencing analyses revealed that transcription of a panel of WNT/β-catenin signaling target genes was reduced predominantly in COLO 320DM cells (Figures 3E and 3G).

**Figure 4:**
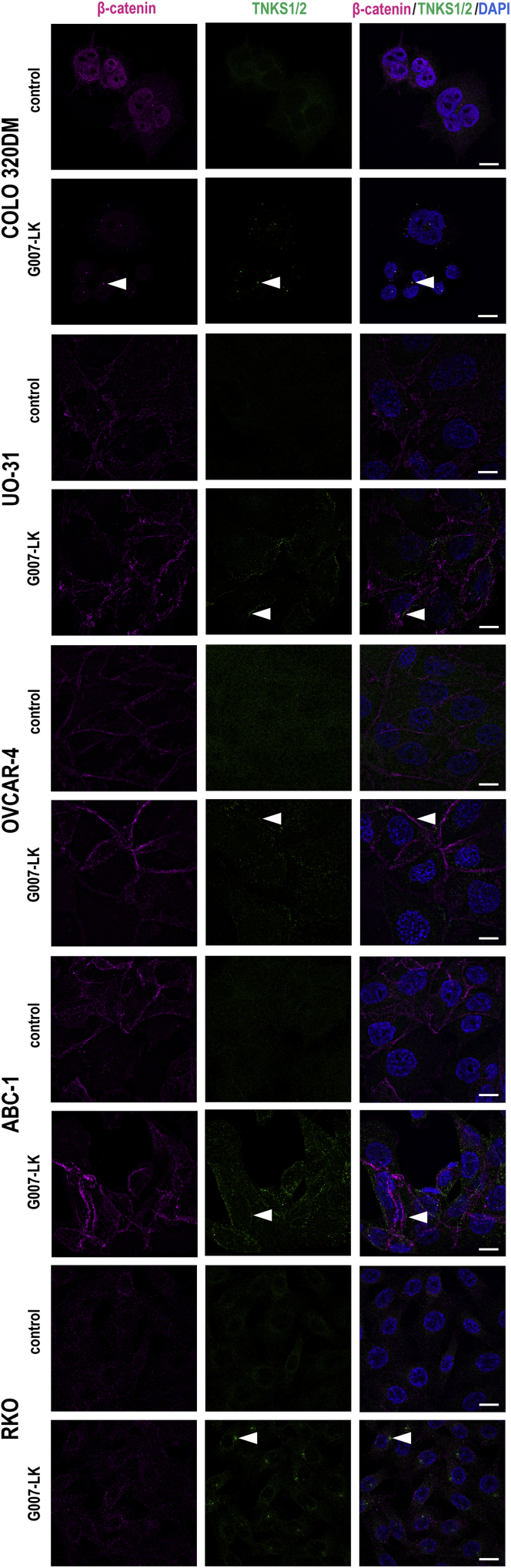
Effect of G007-LK treatment on localization of β-catenin and TNKS1/2 in tumor cell lines Immunofluorescence staining and representative confocal images of β-catenin (magenta) and TNKS1/2 (green) along with nuclear DAPI staining (blue) upon vehicle control (0.01% DMSO) and G007-LK (1 μM) treatment (24 h) of the indicated cell lines. Arrowheads indicate examples of TNKS1/2-containing puncta. Scale bar = 10 μm. See also Figures 3 and S3.

In APC-mutated colorectal cancer cells, TNKSi resulted in the accumulation of cytoplasmic puncta and β*-*catenin degradasomes containing TNKS1/2, AXIN1/2, APC, GSK3β, and β*-*catenin (Thorvaldsen et al., 2015). Hence, to gain further knowledge regarding β*-*catenin degradasome accumulation in the selected cell line panel, structured illumination microscopy imaging was performed to visualize TNKS1/2 and β-catenin upon G007-LK treatment. Decreased accumulation of nuclear β-catenin, and formation of distinct cytoplasmic puncta with colocalized TNKS1/2 and β-catenin (Lau et al., 2013; Thorvaldsen et al., 2015; Waaler et al., 2012), was only observed in APC-mutated COLO 320DM cells with high endogenous WNT/β-catenin signaling activity and expression of AXIN2 protein (Thorvaldsen et al., 2017) (Figure 4). In contrast, β-catenin localization, primarily found in the cell membrane, did not change in the other TNKSi-sensitive cell lines (Figure 4). Instead, TNKS1/2 puncta were found in proximity to the cell membrane after treatment in UO-31, OVCAR-4, and ABC-1 cells (Figure 4). In RKO cells, TNKS1/2 accumulated in juxtanuclear puncta (Figure 4).

In conclusion, the results imply that only COLO 320DM and OVCAR-4 cells are dependent on WNT/β-catenin signaling for sustained cell proliferation, while UO-31 and ABC-1 cells show resistance to β-catenin knockdown. WNT/β-catenin signaling activity is robustly decreased by G007-LK treatment in COLO 320DM cells and modestly in OVCAR-4 and ABC-1 cells. TNKS1/2- and β-catenin-containing puncta are found in the cytoplasm in APC-mutated COLO 320DM cells, but close to the cell membrane in UO-31, OVCAR-4, and ABC-1 cells.

### G007-LK inhibits YAP signaling in the selected cell line panel and all cell lines depend on YAP for sustained proliferation

**Figure 5:**
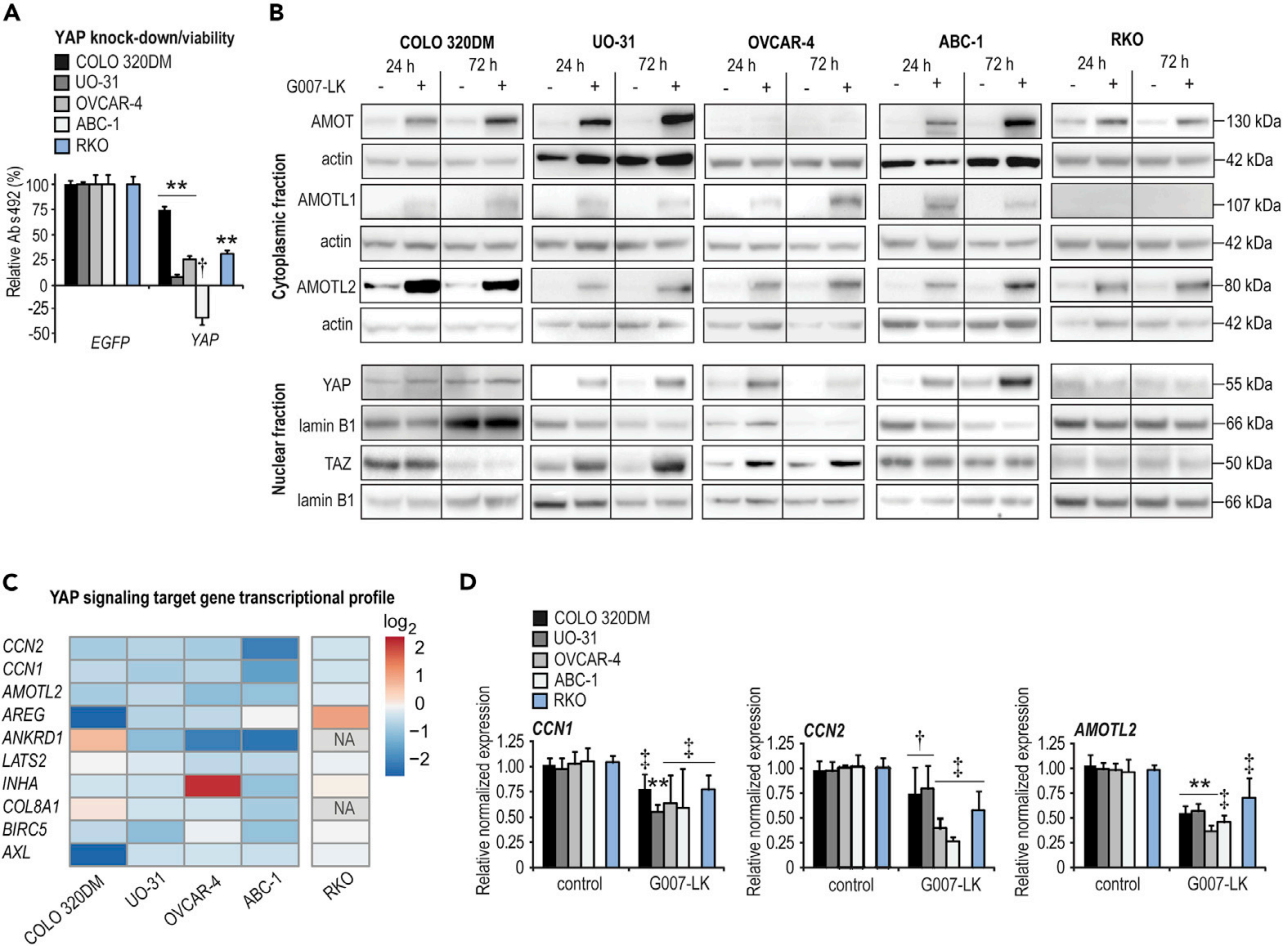
G007-LK inhibits YAP signaling in the selected cell line panel and all cell lines depend on YAP for sustained proliferation (A) Endpoint MTS proliferation assay (Abs_492_) 5–8 days after transfection with siRNA against *YAP* relative to control (100%, *EGFP*) and experiment time 0 values (t_0_, 0%). Mean values ±SD for one representative experiment of minimum three repeated assays with six replicates each are shown. For **A** and **D**, two-tailed t-tests are indicated by ∗∗ (p < 0.01) and Mann-Whitney rank-sum tests are indicated by ‡ (p < 0.01) and † (p < 0.05). (B) Immunoblots of cytoplasmic AMOT, AMOTL1, and AMOTL2 (upper panels) and nuclear YAP and TAZ (lower panels) after 24- or 72-h treatment with G007-LK (1 μM) compared to controls (0.01% DMSO). Actin and lamin B1 document protein loading and representative data from two or more independent experiments are shown. (C) YAP signaling target gene transcription (log_2_) after 24-h treatment with G007-LK (1 μM) compared to controls (0.01% DMSO) (n = 2). NA = not available, no RNA detection. (D) Real-time RT-qPCR analyses of YAP signaling target genes, *CCN1*, *CCN2,* and *AMOTL2*, upon 24-h G007-LK treatment (1 μM) relative to control (0.01% DMSO). Mean values ±SD for combined data from a minimum of two independent experiments with three replicates each are shown. See also Figures 6, S5, and S6.

The IPA core analysis predicted YAP1 as a TNKSi-attenuated upstream regulator, suggestive for decreased YAP signaling in UO-31, OVCAR-4, and ABC-1 cells (Figure 2). To assess whether decreased YAP signaling can impair cell growth, the selected cell panel was transfected with siRNA against *YAP*. Knockdown of *YAP*, to imitate G007-LK-mediated reduction of YAP signaling, resulted in a significant inhibition of cell growth in all cell lines (Figure 5A).

To evaluate the effect of G007-LK on YAP signaling, the selected cell line panel was first examined by Western blot analysis. Treatment of each cell line with G007-LK stabilized AMOT, AMOTL1, and AMOTL2 proteins in both cytoplasmic and nuclear extracts (Figures 5B and S5A), consistent with earlier reports using HEK293T cells (Wang et al., 2015), Nuclear YAP accumulation was enhanced in UO-31, OVCAR-4, and ABC-1 cells, similar to recent observations (Kierulf-Vieira et al., 2020; Waaler et al., 2020b), while no change in nuclear YAP levels were observed in COLO 320DM or RKO cells. Moreover, cytoplasmic YAP was not affected in any cell lines following TNKSi (Figure S5A). The results are in contrast with previous publications showing lowered levels of nuclear YAP upon TNKSi (Wang et al., 2015, 2016) (Figures 5B and S5A).

Although no reduction in nuclear YAP levels was observed in the selected cell line panel subjected to TNKSi, RNA sequencing analyses showed that transcription of a panel of YAP signaling target genes was decreased, in all TNKSi-sensitive cell lines and to a lesser extent in RKO cells (Figure 5C). Real-time qRT-PCR analysis showed reduced transcription of the YAP signaling target genes *CCN1* (previously named *CYR61*), *CCN2* (previously named *CTGF*), and *AMOTL2* in all cell lines (Figure 5D). A moderate and significant reduction in YAP signaling luciferase reporter activity was seen in only COLO 320DM, UO-31, and ABC-1 cells (Figure S5B).

Stabilization of AMOT proteins exposure to TNKSi (Troilo et al., 2016; Wang et al., 2015), and localization of YAP in the degradosome (Azzolin et al., 2014), have previously been described. To obtain additional information regarding the localization of AMOT proteins, YAP, and TNKS1/2 in the selected cell line panel, confocal imaging was next performed.

**Figure 6:**
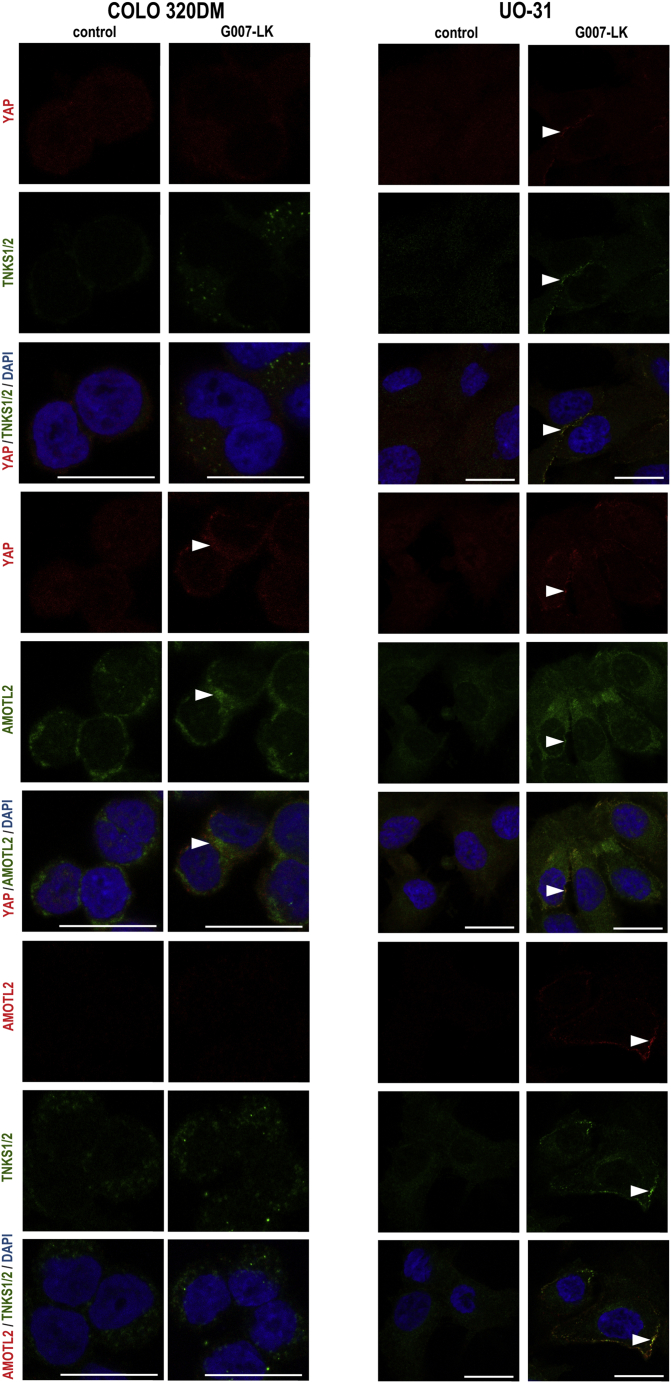
Effect of G007-LK treatment on the localization of YAP, TNKS1/2, and AMOTL2 in tumor cell lines Immunofluorescence staining and representative confocal images of YAP (red) and TNKS1/2 (green), YAP (red) and AMOTL2 (green), or AMOTL2 (red) and TNKS1/2 (green), along with nuclear DAPI staining (blue) upon vehicle control (0.01% DMSO) and G007-LK (1 μM) treatment (24 h) of the indicated cell lines. Red, antimouse antibody used. Green, antirabbit antibody used. Arrowheads indicate colocalizations. Scale bars = 20 μm. See also Figures 5, S5, and S6.

In general, a heterogeneous distribution of nuclear and cytoplasmic YAP, and in addition, a low imaging-detection signal for AMOTL2 in the nuclei, was observed in all cell lines regardless of G007-LK treatment (Figures 6 and S6). However, in UO-31, OVCAR-4 and ABC-1 cells, pairwise colocalization of TNKS1/2-YAP, TNKS1/2-AMOTL2, and AMOTL2-YAP was observed near the cell membrane only after treatment (Figures 6 and S6). In contrast, only AMOTL2-YAP colocalized in COLO 320DM cells (Figure 6). The data propose that TNKSi-induced TNKS1/2-containing puncta can capture AMOTL2-YAP in UO-31, OVCAR-4, and ABC-1 cells, while AMOTL2 sequesters YAP independent of TNKS1/2 in COLO 320DM cells.

In summary, the results show that all cell lines are dependent on YAP signaling for sustained cell growth. G007-LK targets YAP signaling in all cell lines, through a mechanism involving TNKS1/2-mediated and AMOT protein-dependent sequestering and inactivation of transcriptionally active YAP protein.

### G007-LK inhibits PI3K/AKT signaling in ABC-1 cells that depend on PI3K/AKT signaling for sustained cell growth

**Figure 7:**
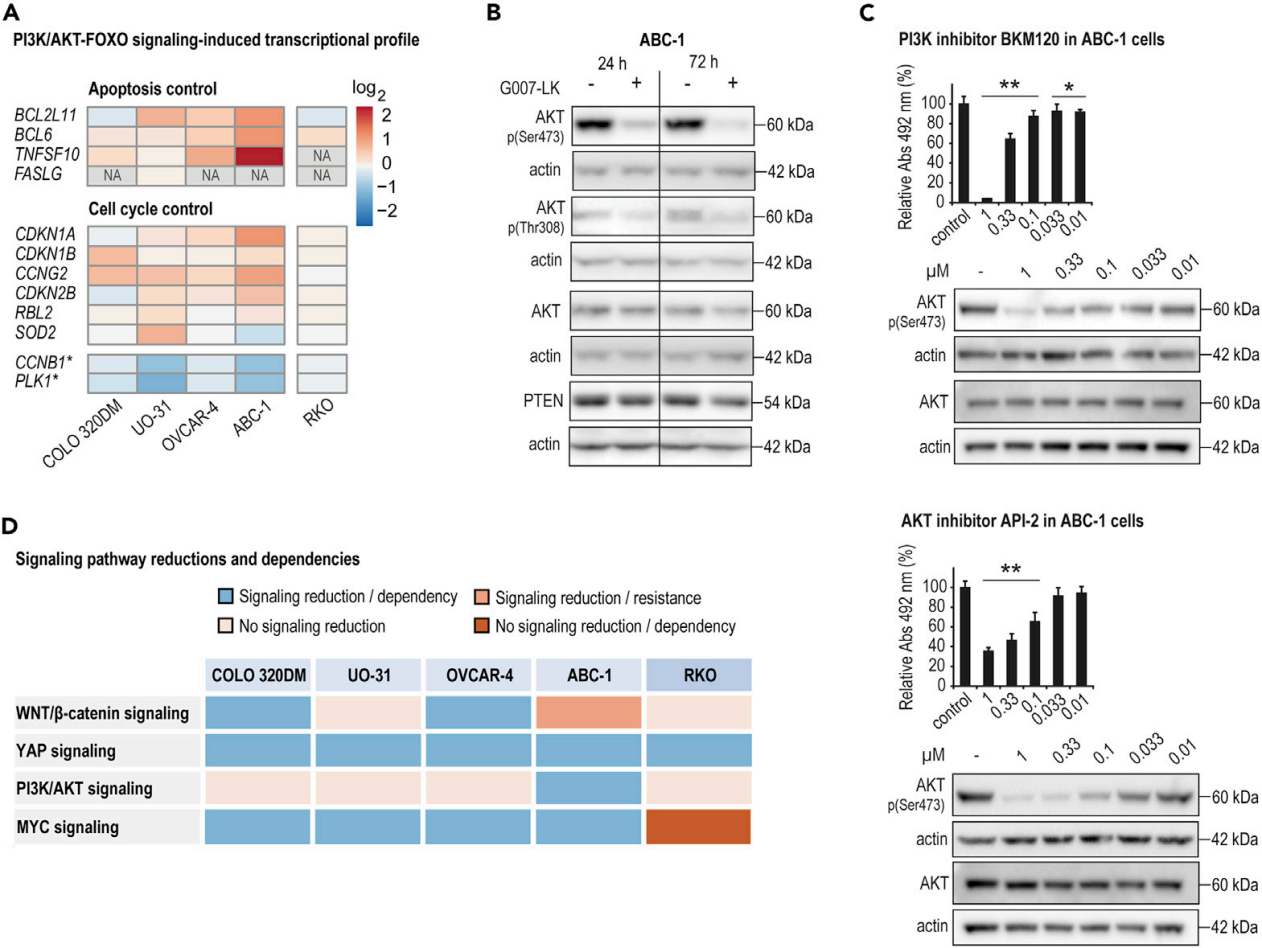
G007-LK inhibits PI3K/AKT signaling in ABC-1 cells that depend on PI3K/AKT signaling for sustained cell growth (A) FOXO-induced signaling target gene transcription (RNA sequencing, log_2_) after 24-h treatment with G007-LK (1 μM) compared to controls (0.01% DMSO) (n = 2). ∗depicts FOXO-inactivated target genes while FOXO-activated genes are nondepicted. NA = not available, no RNA detection. (B) Immunoblots of cytoplasmic active AKT (phospho[Ser473] and phospho[Thr308]), total AKT and PTEN after 24- or 72-h treatment with G007-LK (1 μM) compared to controls (0.01% DMSO) in ABC-1 cells. Actin documents protein loading. Representative data from two or more independent experiments are shown. (C) Left panel, endpoint MTS proliferation assay (Abs_492_ relative to control [100%, 0.01% DMSO] and experiment time 0 values [t_0_, 0%]). Right panel, representative immunoblots of cytoplasmic activate form of AKT (phospho[Ser473]) and total AKT. Upon treatment with indicated concentrations of BKM120 (PI3K inhibitor) and API-2 (AKT inhibitor) in ABC-1 cells for 8 days (MTS analyses) or 24 h (immunoblots). One-way ANOVA tests (Holm-Sidak method versus control) are indicated by ∗∗ (p < 0.01) and ∗ (p < 0.05). For MTS data, mean values ±SD for one representative experiment of minimum two repeated assays with five replicates each are shown. (D) Summary of TNKSi-induced reduction of signaling pathways and dependencies of signaling pathways for sustained cell growth. See also Figure S7.

The IPA core analysis predicted FOXO3 to be a TNKSi-augmented upstream regulator in UO-31 and ABC-1 cells, indicative of TNKSi-dependent decreased PI3K/AKT signaling (Figure 2). However, the RNA sequencing analysis revealed that the transcriptional profile for activated FOXO signaling was most apparently regulated in ABC-1 cells (Figure 7A). Moreover, Western blot analysis was performed to test the effect of G007-LK treatment on PI3K/AKT signaling in the selected cell lines. Reduced presence of the activated and phosphorylated forms of AKT, indicating blocked PI3K/AKT signaling, was only seen in ABC-1 cells (Figures 7B and S7A).

In a previous report, TNKSi stabilized PTEN in colorectal cancer cell lines leading to downregulation of AKT phosphorylation and its signaling activity (Li et al., 2015). In contrast, PTEN was not stabilized in any of the cell lines after treatment, suggesting the presence of a yet-to-be-explored and PTEN-independent mechanism behind TNKSi-induced AKT signaling inactivation (Figure 7B). Earlier publications describe crosstalk signaling between the PI3K/AKT and AMPK signaling pathways (Han et al., 2018; Hawley et al., 2014), and TNKSi has been implicated in regulation of AMPK activation (Li et al., 2019). Hence, the activation status of AMPK in the cell line panel upon G007-LK treatment was evaluated by Western blot analysis. In contradiction with the previous report (Li et al., 2019), our analysis showed that the activated phosphorylated form of AMPK was not upregulated in any of the cell lines by G007-LK exposure (Figure S7B). Instead, AMPK activity was decreased in both UO-31 and ABC-1 cells after TNKSi (Figure S7B). We could therefore not explain the TNKSi-induced AKT inactivation observed only in ABC-1 cells (Han et al., 2018; Hawley et al., 2014). To identify the precise mechanism for TNKSi-induced PI3K/AKT signaling inhibition will require further investigation.

To evaluate if PI3K/AKT signaling is essential for continued cell growth, ABC-1 cells were treated with PI3K and AKT inhibitors. Both inhibitors dose-dependently decreased the active and phosphorylated form of AKT while reducing cell growth (Figure 7C). In conclusion, the data show that G007-LK can target PI3K/AKT signaling in ABC-1 cells that are dependent on PI3K/AKT signaling for continuous cell growth.

## Discussion

Despite more than a decade of research, much remains unclear about the molecular profiles that render tumor cells sensitive or insensitive to the antiproliferative effect of TNKSi. Here, we describe a broad tumor cell line screen, classifying 84% of the cell lines to be TNKSi nonresponders (GI_25_ values > 1 μM G007-LK) and 16% to be TNKSi responders (GI_25_ values < 1 μM G007-LK) including 1.9% to be highly TNKSi-responding tumor cell lines (GI_50_ values < 1 μM G007-LK). >20% of the tumor cell lines originating from the kidney, ovary, stomach, liver, pancreas, and lung were defined as TNKSi responders, suggesting that these types of tumor cell lines are most sensitive to the antiproliferative effect of G007-LK treatment. In general, the result indicates effectiveness of TNKSi against cell growth in subtypes of cancer across several tumor types. ∼85–90% of colon cancer cell lines contain mutations in *APC* resulting in aberrant activation of WNT/β-catenin signaling (Fearon, 2011). Thus, colon cancer was previously regarded a particularly relevant target for TNKSi (Lau et al., 2013). TNKSi-responsiveness in colorectal cancer has been shown to depend on the *APC* mutation genotype (Schatoff et al., 2019). Yet, only two out of 41 colon cancer cell lines tested in our screen displayed GI_25_ values < 1 μM G007-LK.

From the 1.9% highly TNKSi-responding tumor cell lines, a panel of heterogeneous cell lines was selected for further analysis to identify mechanisms coupling TNKSi to attenuated proliferation. In these TNKSi-sensitive cell lines, we used bioinformatics analysis of RNA sequencing data and proteome profiles to characterize TNKSi effects and identified a variety of changes in response signatures. Numerous post-treatment upstream signaling regulators were predicted to be cell-type-dependently controlled by TNKSi according to the IPA core analysis, warranting detailed follow-up studies. While the involvement of additional signaling pathways cannot be excluded, the overall analysis suggests that the main primary events caused by TNKSi in the particular sensitive cell lines are a downregulation of WNT/β-catenin, YAP, and PI3K/AKT signaling pathways followed by decreased MYC expression.

Validation experiments showed that TNKSi (i) blocked WNT/β-catenin signaling in COLO 320DM, OVCAR-4, and ABC-1 cells; (ii) YAP signaling in all cell lines; and (iii) AKT signaling in ABC-1 cells (Figure 7D). Moreover, TNKSi-mediated downregulation of these pathways correlated with lost expression of MYC and CCND1, suggesting that downregulation of these two proteins is a shared hallmark of all tested TNKSi-sensitive cell lines (He et al., 1998; Huh et al., 2019; Kress et al., 2015; Neto-Silva et al., 2010). In line with this notion, functional analyses of TNKSi-mediated cell cycle arrest and apoptosis revealed the induction of a cytostatic effect in all TNKSi-sensitive cell lines, with the exception of ABC-1 cells. In ABC-1 cells, TNKSi stimulated G_1_ cell-cycle arrest, apoptosis and a cytotoxic antiproliferative effect. Moreover, (i) β-catenin knockdown could recapitulate the antiproliferative effect of TNKSi treatment on COLO 320DM and OVCAR-4 cells, (ii) YAP knockdown blocked the growth of all cell lines, whereas inhibition of (iii) PI3K/AKT signaling inhibited the proliferation of only ABC-1 cells (Figure 7D). Notably, combined TNKSi and PI3K/AKT inhibition showed additive antitumor effects in mouse colon cancer models (Arques et al., 2016; Solberg et al., 2018). Collectively, our results suggest that TNKSi-induced inhibition of WNT/β-catenin and YAP signaling, either individually or together, can cause a cytostatic effect, while additional inhibition of PI3K/AKT signaling can provoke an additional cytotoxic effect. Whether similar combinatorial effects against signaling pathways can be projected onto a larger group of tumor cell lines remains to be investigated.

TNKSi stabilized AMOT, AMOTL1, and AMOTL2 in the cytoplasm and the nucleus of all cell lines. In parallel, YAP accumulated in nuclear extracts only in treated UO-31, OVCAR-4, and ABC-1 cells, but not COLO 320DM and RKO cells. YAP signaling target gene expression was reduced in all cell lines. Together, these observations are in line with recent reports that TNKSi induced accumulation of nuclear YAP correlating with reduced YAP target gene expression (Kierulf-Vieira et al., 2020; Waaler et al., 2020b). However, the observations are at odds with earlier reports that TNKSi induced a reduction of nuclear YAP leading to reduced YAP target gene expression (Wang et al., 2015, 2016).

When performing imaging, nuclear AMOTL2 levels were close to, or below the detection threshold, whereas the distribution of nuclear and cytoplasmic YAP was detected regardless of TNKSi. However, in UO-31, OVCAR-4, and ABC-1 cells, puncta containing TNKS1/2-YAP, TNKS1/2-AMOTL2, and AMOTL2-YAP were found near the cell membrane following TNKSi. The results suggest that TNKS1/2-containing β*-*catenin degradasomes (Thorvaldsen et al., 2015) not only can physically interact with YAP, as previously suggested (Azzolin et al., 2014), but also function as complexes containing AMOT proteins (Wang et al., 2015). In contrast, the imaging of COLO 320DM cells revealed the formation of TNKSi-induced cytoplasmic TNKS1/2-puncta that colocalized with β-catenin, indicating β*-*catenin degradasome accumulation (Lau et al., 2013; Thorvaldsen et al., 2015; Waaler et al., 2012). However, in these cells, AMOTL2 and YAP colocalized with each other but not with TNKS1/2. Hence, similar to a previously proposed model (Wang et al., 2015), our observations suggest that AMOT proteins sequester YAP independent of TNKS1/2-containing β*-*catenin degradasomes in COLO 320DM cells. Notably, the APC-mutated cell line COLO 320DM, displaying a high endogenous WNT/β-catenin signaling activity, expresses higher levels of AXIN2 protein compared to the other non-APC-mutated cell lines in the selected panel. In a previous report, loss of expression of AXIN2, but not AXIN1, was associated with disintegration of TNKSi-induced cytoplasmic puncta (Thorvaldsen et al., 2017). The precise mechanism for TNKSi-dependent regulation of YAP signaling, and the association with the β*-*catenin degradasome, is currently under investigation.

In summary, the results provide evidence that TNKSi treatment is effective against subtypes of cancer cell lines across several tumor types. In four identified TNKSi-sensitive cell lines, TNKSi functions by context-dependent targeting of multiple signaling pathways including WNT/β-catenin, YAP and/or PI3K/AKT signaling, followed by loss of MYC expression and the induction of either cytostatic or cytotoxic effects, culminating in impaired tumor cell growth. These findings warrant further comprehensive preclinical and clinical evaluation of TNKSi as monotherapy or combination therapy for cancer.

### Limitations of study

Our study identified several TNKSi-sensitive tumor cell lines, and the downstream in-depth analysis focused on only a small subset of highly sensitive cell lines originating from multiple tissues. These cell lines contain highly diverse oncogenic mutations, gene and protein expression profiles as well as cell signaling pathway activities, and as a consequence, prediction and identification of shared pretreatment and post-treatment markers was influenced. Numerous TNKSi-induced changes in activities of signaling pathways, in addition to WNT/β-catenin, YAP, and PI3K/AKT signaling pathways, were predicted and detailed follow-up studies are clearly needed. The experiments provide only a limited description of TNKSi-dependent regulation of YAP signaling, and the results contradict with previous descriptions of TNKSi-regulated PTEN/PI3K/AKT and AMPK signaling pathways activities, indicating that further research is needed to identify the precise mechanisms involved.

## STAR★Methods

### Key resources table

**Table:** 

REAGENT or RESOURCE	SOURCE	IDENTIFIER
**Antibodies**		

MYC	Cell Signaling Technology	Cat#5605; RRID: AB_1903938
CCND1	Abcam	Cat# ab16663; RRID: AB_443423
TNKS1/2	Santa Cruz Biotechnology	Cat#sc-8337; RRID: AB_661615
AXIN1	Cell Signaling Technology	Cat#2087; RRID: AB_2274550
AXIN2	Cell Signaling Technology	Cat#2151; RRID: AB_2062432
non-phospho (active) β-catenin	Cell Signaling Technology	Cat#8814; RRID: AB_11127203
phospho β-catenin (Ser33/37/Thr41)	Cell Signaling Technology	Cat#9561; RRID: AB_331729
β-catenin	BD Biosciences	Cat#610153; RRID: AB_397554
GSK3β	Cell Signaling Technology	Cat#12456; RRID: AB_2636978
phospho-GSK3β (Ser9)	Cell Signaling Technology	Cat#9323; RRID: AB_2115201
AMOT	Santa Cruz Biotechnology	Cat#sc-166924; RRID: AB_10609353
AMOTL1	Thermo Fisher Scientific	Cat#PA5-42267; RRID: AB_2606805
AMOTL2	Thermo Fisher Scientific	Cat#PA5-78770; RRID: AB_2745886
AMOTL2	Santa Cruz Biotechnology	Cat#sc-398261; RRID:N/A
YAP	Santa Cruz Biotechnology	Cat#sc-101199; RRID: AB_1131430
TAZ	Sigma Aldrich	Cat#HPA007415; RRID: AB_1080602
AKT	Cell Signaling Technology	Cat#9272; RRID: AB_329827
phospho-AKT (Ser473)	Cell Signaling Technology	Cat#4060; RRID: AB_2315049
phospho-AKT (Thr308)	Cell Signaling Technology	Cat#9275; RRID: AB_329828
PTEN	Cell Signaling Technology	Cat#9552; RRID: AB_10694066
AMPKα	Cell Signaling Technology	Cat#2793; RRID: AB_915794
phospho-AMPKα (Thr172)	Cell Signaling Technology	Cat#2535; RRID: AB_331250
Actin	Sigma Aldrich	Cat#A2066; RRID: AB_476693
lamin B1	Abcam	Cat#ab16048; RRID: AB_10107828

**Bacterial and virus strains**		

SuperTOP-luciferase (WNT/β-catenin signaling pathway reporter with 7xTCF binding sites: SuperTOPflash)	Gift from Dr. Vladimir Korinek)	N/A
FOPflash (negative control reporter with mutated TCF binding sites: SuperFOPflash-luciferase)	Gift from Dr. Vladimir Korinek)	N/A
8xGTIIC-luciferase (Hippo and YAP signaling pathway reporter)	Addgene, provided by Dr. Stefano Piccolo	RRID: Addgene_34615
*Renilla* luciferase (pRL-TK)	Promega	Cat#E2241
7TFP (7xTcf-FFluc//SV40-PuroR)	Addgene, provided by Dr. Roel Nusse	RRID: Addgene_24308
pMD2.G (VSV-G envelope expressing plasmid)	Addgene, provided by Dr. Didier Trono	RRID: Addgene_12259
psPAX2 (lentiviral packaging plasmid)	Addgene, provided by Dr. Didier Trono	RRID: Addgene_12260

**Chemicals, peptides, and recombinant proteins**		

G007-LK	ChemRoyal and Mercachem	N/A
BKM120	Chemietek	Cat#CT-BKM120
API-2	Tocris Bioscience	Cat#2151
NP40 cell lysis buffer	Invitrogen	Cat#FNN0021
RIPA lysis buffer	Thermo Fisher Scientific	Cat#89901
RPMI 1640 Medium for SILAC	Thermo Fisher Scientific	Cat#11586861
L-Lysine-2HCl, 13C6 for SILAC	Thermo Fisher Scientific	Cat#11854191
L-Arginine-HCl, 13C6, 15N4 for SILAC	Thermo Fisher Scientific	Cat#11546871
Dialyzed FBS for SILAC	Thermo Fisher Scientific	Cat#11506871

**Critical commercial assays**		

CellTiter 96® AQueous Non-Radioactive Cell Proliferation Assay (MTS)	Promega	Cat#G5421
Lipofectamine 2000	Invitrogen	Cat#11668019
Annexin V-FITC Apoptosis Detection Reagent	Abcam	Cat#ab14082
GenEluteTM Mammalian Total RNA Miniprep Kit	Sigma-Aldrich	Cat#RTN350
SuperScript^TM^ VILO cDNA Synthesis Kit	Invitrogen	Cat#11754250
PierceTM BCA Protein Assay Kit	Pierce Biotechnology	Cat#23227
Dual-Luciferase Reporter Assay	Promega	Cat#E1980
Deposited data		
RNASeq dataset	GEO database	GSE162648
R-code for analysis	Github	https://github.com/MartinFStrand/TNKSiRes
Proteomics dataset	ProteomeXchange	PXD022908

**Experimental models: Cell lines**		

COLO 320DM	American Type Culture Collection (ATCC)	Cat#ATCC® CCL220™; RRID: CVCL_0219
UO-31	NCI-60 Human Tumor Cell Lines Screen/ National Cancer Institute (NCI-DTP)	Cat#UO-31; RRID:CVCL_1911
OVCAR-4	NCI-60 Human Tumor Cell Lines Screen/ National Cancer Institute (NCI-DTP)	Cat#OVCAR-4; RRID:CVCL_1627
ABC-1	Japanese Collection of Research Bioresources Cell Bank (JCRB)	Cat#JCRB0815; RRID:CVCL_1066
RKO	American Type Culture Collection (ATCC)	Cat#ATCC® CRL2577™; RRID:CVCL_0504

**Oligonucleotides**		

*MYC* (esiRNA)	Sigma Aldrich	Cat#EHU021051
*CTNNB1* (esiRNA)	Sigma Aldrich	Cat#EHU139421
*YAP1* (esiRNA)	Sigma Aldrich	Cat#EHU113021
*EGFP* (esiRNA)	Sigma Aldrich	Cat#EHUEGFP
*MYC* (TaqMan probe)	Applied Biosystems	Catt#Hs00153408_m1
*CCND1* (TaqMan probe)	Applied Biosystems	Cat#Hs00765553_m1
*AXIN2* (TaqMan probe)	Applied Biosystems	Cat#Hs00610344_m1
*CYR61* (*CCN1*, TaqMan probe)	Applied Biosystems	Cat#Hs00155479_m1
*CTGF* (*CCN2*, TaqMan probe)	Applied Biosystems	Cat#Hs01026927_g1
*AMOTL2* (TaqMan probe)	Applied Biosystems	Cat#Hs01048101_m1
*GAPDH* (TaqMan probe)	Applied Biosystems	Cat#Hs02758991_g1

**Software and algorithms**		

Sigma Plot® 12.5	Systat Software Inc.	https://systatsoftware.com/products/sigmaplot/
ControlFreak	Contchart software	https://contchart.com/outliers.aspx
XLfit SOAPaligner/SOAP2	IDBS	https://www.idbs.com/excelcurvefitting/
Software for short oligonucleotide alignment		https://github.com/ShujiaHuang/SOAPaligner
NOISeq – R package for exploratory analysis and differential expression for RNA-seq data	(Tarazona et al., 2011, 2015)	https://www.bioconductor.org/packages/release/bioc/html/NOISeq.html
Pheatmap – R package for heatmap generation	Raivo Kolde	https://CRAN.R-project.org/package=pheatmap
Ingenuity Pathway Analysis (IPA version 01-10)	Qiagen	https://digitalinsights.qiagen.com/products-overview/discovery-insights-portfolio/analysis-and-visualization/qiagen-ipa/
GenVisR (R package for mutation waterfall plots)	Bioconductor	http://bioconductor.org/packages/release/bioc/html/GenVisR.html
MaxQuant (MaxQuant version 1.3.2.8)	Max-Planck-Institute of Biochemistry	https://www.maxquant.org/

**Other**		

IncuCyte	Essen BioScience	Cat# FLR30140
GloMax®-Multi Detection System	Promega	Cat# E7031

### Resource availability

#### Lead contact

Further information and requests for resources and reagents should be directed to and will be fulfilled by the Lead Contact Jo Waaler (jo.waaler@rr-research.no).

#### Materials availability

Materials generated in this study can be made available upon request to the Lead Contact.

#### Data and code availability

Datasets and codes generated during this study are available at repositories indicated in the key resources table. Raw and processed mRNA-sequencing data have been deposited in NCBI's Gene Expression Omnibus (Edgar et al., 2002) and are accessible through GEO Series accession number GSE162648 (https://www.ncbi.nlm.nih.gov/geo/query/acc.cgi?acc=GSE162648). The mass spectrometry proteomics data have been deposited to the ProteomeXchange Consortium via the PRIDE (Perez-Riverol et al., 2019) partner repository with the dataset identifier PXD022908. The R-code used for analysis and visualization is available at https://github.com/MartinFStrand/TNKSiRes. Original and source data for the mutation analysis, for the human cancer cell lines COLO320DM, OVCAR-4, U031, ABC-1 and RKO, as indicated in Table S2, is available from Cancer Cell Line Encyclopedia (CCLE; COLO320_LARGE_INTESTINE, ABC1_LUNG, UO31_KIDNEY, OVCAR4_OVARY, RKO_LARGE_INTESTINE), Catalogue of Somatic Mutations in Cancer (COSMIC; COLO-320-HSR [COSS910569], ABC-1 [COSS906791], OVCAR-4 [COSS905990], UO-31 [COSS905981] and RKO [COSS909698]) and canSAR (Coker et al., 2019).

### Experimental model and subject details

#### Cell lines and cell culture

The human cancer cell lines COLO 320 DM (colorectal adenocarcinoma, ATCC® CCL220™) and RKO (colon carcinoma, ATCC® CRL2577™) were obtained from the American Type Culture Collection (ATCC). ABC-1 cells (lung adenocarcinoma, JCRB0815) were obtained from the Japanese Collection of Research Bioresources Cell Bank (JCRB). UO-31 (renal cell carcinoma) and OVCAR-4 cells (ovarian adenocarcinoma), from the NCI-60 Human Tumor Cell Lines Screen, were provided by the National Cancer Institute (NCI). ABC-1 and RKO were cultured in Eagle's Minimum Essential Medium (EMEM, 30-2003, ATCC), while COLO 320DM, OVCAR-4 and UO-31 cells were cultured in RPMI-1640 medium (R8758, Sigma-Aldrich). Both media contained 10% Fetal Bovine Serum (FBS, 10270-106, Gibco) and 1% Penicillin-Streptomycin (P4333, Sigma-Aldrich). Cells were cultured at 37 ^o^C in humidified cell incubators with 5% CO_2_. The cell cultures were kept below 20 passages (∼10 weeks) and routinely monitored (upon thawing and monthly) for Mycoplasma infections with MycoAlert Mycoplasma detection kit (Lonza). All cell lines were authenticated by short tandem repeat profiling to confirm their identity (Eurofins).

### Method details

#### Human tumor cell line anti-proliferative screens

Compound screening and data analysis carried out by Genentech was performed similar to a previous description (Haverty et al., 2016). Briefly, a collection of cancer cell lines obtained from a variety of academic and commercial sources, such as ATCC and Leibniz Institute DSMZ-German Collection of Microorganisms and Cell Cultures, was used. Cell line identity was routinely verified by high-throughput single nucleotide polymorphism genotyping using multiplexed assays (Yu et al., 2015). All cell lines were cultured using standard tissue culture techniques and maintained in RPMI-1640 (31800, GIBCO), 2 mM glutamine (Kyowa Hakko Bio), 10% FBS (F4135, Sigma) for suspension cell lines, and 5% FBS for adherent cell lines, in a humidified incubator maintained at 37°C and 5% CO_2_. Cells were plated in 384-well plates (353962, Corning) at optimal seeding density to achieve 75% confluency at 96 hours monitored using Incucyte for live cell imaging (4647, Essen Bioscience). Optimal seeding for suspension and mix suspension/adherent cell lines was determined by 75% maximal signal at 96 hours using CellTiter-Glo® Luminescent Cell Viability Assay (G7573, Promega). The day after, cell culture medium was changed to medium containing nine drug concentrations (using three to four replicates) or vehicle control (dimethylsulfoxide, DMSO, D2650, Sigma). After 72 hours, 25 μL CellTiter-Glo® reagent was added to the wells and luminescence readout was measured using a 2104 EnVision Multilabel Plate Reader (2105-0010, PerkinElmer). Data were processed using R (R Core Team, 2012) and a Genentech-developed analysis package (singleAgentPlots, Dr. Richard Bourgon). Absolute GI_25_ and GI_50_ values were calculated relative to the corresponding vehicle control.

G007-LK was screened against the NCI-60 tumor cell line panel using their standard protocol: Briefly, all cell lines were grown in RPMI 1640 medium containing 5% FBS and 2 mM L-glutamine at 37 °C and 5% CO_2_. 5,000-40,000 cells/well were seeded in 96-well plates depending on the cell’s doubling speeds. The day after, cell culture medium was changed to medium containing five drug concentrations plus control. After an additional 48 hours, adherent cells were fixed *in situ* by the addition of 50 μl of cold 50 % (w/v) TCA (final concentration, 10 %) and incubated for 60 minutes at 4 °C. The supernatant was discarded, and the plates were washed five times with water and air dried. Sulforhodamine B (SRB) solution (100 μl) at 0.4 % (w/v) in 1 % acetic acid was added to each well, and the plates were incubated for 10 minutes at room temperature. After staining, cells were rinsed five times with 1 % acetic acid and the plates were air dried. Bound SRB was subsequently solubilized with 10 mM trizma base, and the absorbance was read on an automated plate reader at a wavelength of 515 nm. For suspension cells, the methodology was the same except that the cells were allowed to settle to the bottom of the well before gently adding 50 μl of 80 % TCA (final concentration 16 %). Using seven absorbance measurements (time zero [Tz], control growth [C], and test growth in the presence of five drug concentrations [Ti]), the percentage growth inhibition (GI_25_ and GI_50_ values) was calculated for each drug concentration using the formula: (1- [Ti-Tz]/[C-Tz]) x 100.

#### Treatment with small-molecule inhibitors

All small-molecule inhibitors were dissolved in DMSO (D8418, Sigma-Aldrich) and kept as 10 mM stocks at 4 ^o^C. General protocol for treatment: Cells were seeded one day before treatment to reach ∼20 or ∼80% confluence for a 72 hour or a 24 hour treatment, respectively. The medium was changed to medium containing vehicle (0.01% DMSO), 1 μM or various doses of the tankyrase inhibitor G007-LK (Mercachem), PI3K inhibitor BKM120 (CT-BKM120, Chemietek) or AKT inhibitor API-2 (2151, Tocris Bioscience).

#### siRNA transfection

Cells were seeded in 6-well plates to reach 50-60% confluence the day after, when the cells were transfected (Lipofectamine 2000, 11668019, Invitrogen) with esiRNA against *MYC* (50 nM, EHU021051), *CTNNB1* (50 nM, EHU139421) or *YAP1* (25 nM, EHU113021), using esiRNA against *EGFP* (50 or 25 nM, EHUEGFP) as control (all Sigma-Aldrich). The next day, the cells were trypsinized (T392, Sigma-Aldrich) and seeded in 96-well plates for proliferation assays, or in 6-well plates for 48 additional hours for preparing protein extracts, cell cycle analyses and apoptosis assays.

#### Proliferation assays

5,000 (COLO 320DM), 2,500 (UO-31, OVCAR-4, ABC-1) or 1,000 (RKO) cells/well were seeded in 96-well plates in at least 6 replicates for each treatment tested, also for seeding of esiRNA transfected cells. For samples for treatment with small-molecule inhibitors, the cell culture medium was changed the day after to contain various doses of the indicated inhibitors or 0.01% DMSO. The plates were incubated at 37 °C or in an IncuCyte (FLR30140, Essen BioScience) for real-time monitoring of cell confluency. At experiment endpoint (80-100% confluency after 5-8 days of cell growth), the cells were incubated for 1 hour at 37°C with CellTiter 96® AQueous Non-Radioactive Cell Proliferation Assay (MTS, G5421, Promega) according to the supplier’s recommendations. Abs_492_ was measured (Wallac 1420 Victor2 Microplate Reader, Perkin Elmer) and compared the initial Abs_492_ values (t0) using the following formula to determine single well values relative to the vehicle or *EGFP* esiRNA controls: (sample A_492_ - mean A_492 t0_)/(mean A_492_ [for 0.01% DMSO controls] - mean A_492 t0_) ×100.

#### Colony assays

500 cells/well were seeded in 6-well plates. The day after, cell culture medium with 10% FBS was changed to contain 1 μM G007-LK or vehicle (0.01% DMSO) and the plates were incubated at 37 °C for 7-11 days without replacing the medium. Colonies were stained and fixed (0.2% methylene blue [M9140] in methanol **[**82762], both Sigma-Aldrich), washed with PBS, and enumerated using a colony counter (Scienceware). For UO-31 cells, colony confluence was measured at experiment end using IncuCyte.

#### Cell cycle and apoptosis assays

After treatment with vehicle, G007-LK or esiRNA, both cells in suspension and trypsinized adherent cells were collected to form a single cell suspension. Cell cycle assay: The cells were vortexed vigorously while drop-wisely adding ice cold 70% ethanol followed by incubation at -20 ^o^C for at least 12 hours. The cells were then pelleted by centrifugation, washed in PBS and re-suspended in PBS containing propidium iodide (20 μg/ml, P4170, Sigma-Aldrich], RNaseA (200 μg/ml, R5503, Sigma-Aldrich) and Triton X-100 (0.1%, T8787, Sigma-Aldrich) for 15 minutes at 37 ^o^C before cell cycle analysis. Apoptosis Assay: The cells were pelleted by centrifugation and incubated for 10 minutes at 37 ^o^C in Annexin V-FITC Apoptosis Detection Reagent (ab14082, Abcam). PI positive and Annexin V-FITC positive cells were quantified by flow cytometry using an Attune Acoustic Focusing Cytometer (Applied Biosystems).

#### RNA isolation and real-time qRT-PCR

Total mRNA was isolated from treated cells using GenEluteTM Mammalian Total RNA Miniprep Kit (RTN350, Sigma-Aldrich) and quantified using a Nanodrop 2000c spectrophotometer (Thermo Scientific). cDNA was synthesized with SuperScript^TM^ VILO cDNA Synthesis Kit (11754250, Invitrogen) and real-time qRT-PCR (TaqMan®Gene Expression system, Applied Biosystems) was performed using Viia7 Real-Time PCR System (Applied Biosystems). The following probes (all from Applied Biosystems) were used: *MYC* (Hs00153408_m1), *CCND1* (Hs00765553_m1), *AXIN2* (Hs00610344_m1), *CYR61* (*CCN1*, Hs00155479_m1), *CTGF* (*CCN2*, Hs01026927_g1), *AMOTL2* (Hs01048101_m1) and *GAPDH* (Hs02758991_g1).

#### Western blot analysis

Cells treated with small-molecule inhibitors or esiRNA were washed with PBS and lysed in NP40 buffer (FNN0021, Invitrogen) containing protease inhibitors (cOmplete™ Protease Inhibitor Cocktail, 4693116001, Roche). Pelleted nuclei were separated from the cytoplasmic-cell membrane supernatant fractions. RIPA lysis buffer (89901, Thermo Fisher Scientific), containing phosphatase (4906845001) and protease inhibitors (4693116001, both Sigma-Aldrich) was added to the nuclei followed by sonication (Bioruptor®Plus, Diagenode). Protein concentrations were measured using PierceTM BCA Protein Assay Kit (23227, Pierce Biotechnology). The protein extracts were loaded to gels (NuPAGE® Novex 3-8% Tris-Acetate Gel or 4-12% Bis-Tris Gel, Invitrogen), separated and transferred onto PVDF membranes (Immobilon-PSQ PVDF Membrane, Millipore). The following primary antibodies were used to probe the membranes: MYC (5605, Cell Signaling Technology), CCND1 (ab16663, Abcam), Tankyrase-1/2 (TNKS1/2, H-350, sc-8337, Santa Cruz Biotechnology), AXIN1 (2087, Cell Signaling Technology), AXIN2 (2151, Cell Signaling Technology), non-phospho (active) β-catenin (8814, Cell Signaling Technology), phospho β-catenin (Ser33/37/Thr41)(9561, Cell Signaling Technology), β-catenin (610153, BD Biosciences), GSK3β (12456, Cell Signaling Technology), phospho-GSK3β (Ser9)(9323, Cell Signaling Technology), AMOT (sc-166924, Santa Cruz Biotechnology), AMOTL1 (PA5-42267, Thermo Fisher Scientific), AMOTL2 (PA5-78770, Thermo Fisher Scientific), YAP (sc-101199, Santa Cruz Biotechnology), TAZ (HPA007415, Sigma-Aldrich), AKT (9272, Cell Signaling Technology), phospho-AKT (Ser473)(4060, Cell Signaling Technology), phospho-AKT (Thr308)(9275, Cell Signaling Technology), PTEN (9552, Cell Signaling Technology), AMPKα (2793, Cell Signaling Technology) and phospho-AMPKα (Thr172)(2535, Cell Signaling Technology). Actin (A2066, Sigma-Aldrich) and lamin B1 (ab16048, Abcam) were used as loading controls.

#### Luciferase reporter assays

The following plasmids were used for transient co-transfections: SuperTOP-luciferase (WNT/β-catenin signaling pathway reporter with 7xTCF binding sites: SuperTOPflash, gift from V. Korinek), FOPflash (negative control reporter with mutated TCF binding sites: SuperFOPflash-luciferase, gift from V. Korinek), 8xGTIIC-luciferase (Hippo and YAP signaling pathway reporter: 34615, Addgene, provided by S. Piccolo) and *Renilla* luciferase (E2241, pRL-TK, Promega). FuGENE*®* HD (Promega) was used for all co-transfections. For co-transfections using SuperTOPflash or FOPflash and *Renilla* luciferase: On day one, cells were seeded in 48-well plates to reach 50-60% confluency on day two for co-transfections (0.23 μg luciferase reporter and 0.02 μg *Renilla* luciferase) followed by 24 hours cultivation. For 8xGTIIC-luciferase and *Renilla* luciferase co-transfections: Cells were seeded in 10-cm dishes on day one to reach 50-60% confluency on day two for co-transfections (16.5 μg 8xGTIIC-luciferase and 3 μg *Renilla* luciferase). On day three, the cells were trypsinized. 40,000 and 10,000 cells were seeded in 96-well plates for 24 and 72 hours G007-LK treatments starting on day four, respectively. For generation of stable cell lines: Lentivirus packed with 7TFP (7xTcf-FFluc//SV40-PuroR, #24308, Addgene, provided by R. Nusse), pMD2.G (VSV-G envelope expressing plasmid, #12259, Addgene, provided by D. Trono) and psPAX2 (lentiviral packaging plasmid, #12260, Addgene, provided by D. Trono) were used for creating lentiviral particles. All cell lines were transduced and thereafter treated with 0.5-5 μg/ml Puromycin (P9620, Sigma-Aldrich) for selection. Next, the cells lines were transduced using lentiviral particles expressing *Renilla* luciferase (LVP370, Amsbio) followed by selection using 300-1,000 μg/ml Geneticin (11558616, Fisher Scientific). 40,000 and 10,000 cells were seeded in 96-well plates for 24 and 72 hours G007-LK treatments starting the day after, respectively. At experiment end, the cells were lysed and the luciferase activities were measured using Dual-Luciferase Reporter Assay (E1980, Promega) and GloMax®-Multi Detection System (E7031, Promega). XLfit (Idbs) was used to calculate the 50% inhibitory concentration (IC_50_) using the Langmuir Binding Isotherm formula.

#### Structured illumination and confocal microscopy

Similar to our previous protocol (Waaler et al., 2020b), cells grown on coverslips pre-coated with poly-L-lysine (sc-286689, Santa Cruz Biotechnology) were fixed in 4% paraformaldehyde (P6148, Sigma-Aldrich) for 15 minutes at room temperature and permeabilized with 0.1% Triton-X100/PBS (T8787, Sigma-Aldrich, 15 minutes at room temperature). Incubation with primary antibodies (24 hours at 4 °C) was followed by incubations with secondary antibodies (1 hour at room temperature), both diluted in PBS with 4% bovine serum albumin. Nuclear counterstaining was performed with DAPI (D9542, Sigma-Aldrich, 1 μg/mL, 5 minutes at room temperature) and coverslips were mounted in ProLong Diamond Antifade Mountant (Thermo Fisher Scientific). The following primary antibodies were used: β-catenin (610153, 1:500, BD Biosciences), Tankyrase-1/2 (H-350, sc-8337, 1:50, Santa Cruz Biotechnology), YAP (sc-101199, 1:200, Santa Cruz Biotechnology), AMOTL2 (sc-398261, 1:50, Santa Cruz Biotechnology) and AMOTL2 (PA5-78770, 1:50, Thermo Fisher Scientific). Secondary antibodies used (both from Thermo Fisher Scientific, 1:500): Anti-rabbit IgG Alexa488 (A-21206) and anti-Mouse IgG Alexa594 (A-11005). Structured illumination microscopy (SIM) images were acquired on a Zeiss Elyra PS1 microscope system using standard filters sets and laser lines with a Plan-APOCHROMAT 63× 1.4 NA oil objective. SIM imaging was performed using 5 grid rotations with the 0.51 μm grid for 20 Z planes with 0.184 nm spacing between planes. SIM images were reconstructed with the following “Method” parameters in the ZEN black software (MicroImaging, Carl Zeiss): Processing: Manual, Noise Filter: -5, SR Frequency Weighting: 1, Baseline Cut, Sectioning: 100/83/83, Output: SR-SIM, PSF: Theoretical. The SIM images are displayed as maximum intensity projections rendered from all Z planes. Confocal microscopy was performed on a Zeiss Meta 700 laser scanning confocal microscope using standard filters sets and laser lines with a 63× oil immersion objective, images were acquired as Z-stacks using the Zen software package (Zeiss) with 0.56 μm spacing between stacks. The confocal images were analyzed using the Fiji software tool (Schindelin et al., 2012). Confocal images were displayed as Z-stacks in the nucleous region.

#### RNA sequencing and alignment

mRNA from all cell lines were isolated then treated with DNase I (AMPD1-1KT, Sigma-Aldrich) before 4 μg mRNA was sent to BGI Tech Solutions Company (Hong Kong) for RNA sequencing according to their standard protocol. Briefly, total RNA samples were enriched using oligo(dT) magnetic beads followed by fragmentation (about 200 bp). Then double-stranded cDNA was synthetized and purified, before end reparation, ligation of sequencing adaptors and enrichment by PCR amplification. Quality control and quantification was performed using Agilent 2100 Bioanalyzer and ABI StepOnePlus Real-Time PCR System prior to library sequencing via Illumina HiSeq™ 2000. For analysis, clean reads were mapped to the reference gene set (hg19) using SOAPaligner/SOAP2 with no more than 2 mismatches allowed in the alignment. Gene expression level was calculated using RPKMs (reads per kb transcript per million mapped reads).

#### Bioinformatics

NOISeq analysis (Tarazona et al., 2011, 2015) was used by the BGI Tech Solutions Company to identify differentially expressed genes (DEGs) between treatment (1 μM G007-LK) and control (0.01% DMSO) groups (both n =2). Genes with a differential expression (DE) probability > 0.8 were defined by default as DEGs. The DE probability is calculated in NOISeq by comparing the log_2_ ratio of the two conditions (M) and the value of difference between conditions (D) against the noice distribution, and a higher probability value corresponds to a higher significance (inverse of a normal P value)(Tarazona et al., 2011). As several applications use P values as a default, the DE probability was transformed using the following formula: 1-Probability. The NOISeq DE probability calculation performs well with regards to false discovery rate (FDR) compared to other methods, and thus the inverse of the DE-probability (1-Probability) may be used as the equivalent of a corrected P value. Volcano plots and heatmaps (using pheatmap, https://CRAN.R-project.org/package=pheatmap) were generated in R (version 3.6.1). For each cell line, a list of DE genes, including log_2_ fold change and transformed probability values (1-Probability), was analyzed using Ingenuity Pathway Analysis (IPA) version 01-10 (Qiagen). For each cell line, log_2_ fold change and transformed probability values from the DEGs analysis were uploaded into IPA and analyzed for upstream regulators using the core analysis function. The core analysis was performed with the Ingenuity Knowledge Base (genes only) reference set and direct relationships, with no filters set for node types, data sources, confidence, species, tissues & cell lines or mutations.

#### Mutation analysis

Mutation datasets for the cell lines were downloaded from the CCLE, COSMIC and CANSAR databases and screened for a set of 299 driver mutations previously identified (Bailey et al., 2018) and plotted as a waterfall plot using the GenVisR package (Skidmore et al., 2016) in R.

#### Proteomics

All cell lines were treated in duplicates with 1 μM G007-LK or 0.01% DMSO for 24 hours. In parallel, all cell lines were cultured in heavy medium for SILAC labeling (11586861, RPMI 1640 Medium for SILAC; 11854191, L-Lysine-2HCl, 13C6 for SILAC; 11546871, L-Arginine-HCl, 13C6, 15N4 for SILAC; 11506871, Dialyzed FBS for SILAC). After cultivation, pelleted cells were dissolved in 0.1% ProteaseMax Surfactant (V2071, Promega) in 50 mM NH_4_HCO_3_ (A6141, Sigma-Aldrich). Samples were heated at 95 °C for 15 minutes and then sonicated for 15 minutes. After 3 freeze and thaw cycles, the samples were vigorously mixed and passed a syringe (0.9 x 2.5 mm) to dissolve DNA, and heating and sonication was repeated as described above. Then, the samples were centrifuged at 14,000 *g* before measuring the protein concentration of the supernatant by DirectDetect (DDHW00010-W, Millipore). For each cell line, 100 μg protein was transferred to new vials and mixed with 100 μg of protein from corresponding SILAC labeled cells. Each sample was mixed with 40 μl 0.2% ProteaseMax Surfactant in 50 mM NH_4_HCO_3_, followed by further dilution with 147 μl 50 mM NH_4_HCO_3_. Proteins were next reduced with 5 mM 1,4-dithiothreitol (D9779-10G, Sigma-Aldrich) at 56 °C for 20 minutes, and subsequent digestion with 4 μg trypsin (V5111, Promega) overnight at 37 °C. Peptides were desalted on a C_18_ StageTip made with three layers of C_18_ Empore Extraction disks (98060402140, 3M), and eluted with 80% acetonitrile (ACN, 34851, Sigma-Aldrich)/0.1% formic acid (FA, 533002, Merck). The eluate was dried on a SpeedVac (5305000100, Eppendorf) until the volume reached approximately 3 μl. The sample was next reconstituted to a total volume of 14 μl in 0.1% FA. nLC-MS/MS analysis was performed on a nEASY-LC system coupled to a Q-Exactive Plus mass spectrometer (IQLAAEGAAPFALGMBDK, both Thermo Fisher Scientific). A 25 cm EasySpray column (C18, 2 μm beads, 100 Å, 75 μm inner diameter, S802, Thermo Electron) was used to separate peptides, using a 120 minutes gradient up to a concentration of 30%. Solvent A (0.1% FA) and solvent B (100% ACN with 0.1% FA) was used with a flow rate of 0.3 μl/minutes. The mass spectrometer was operated in a data-dependent mode with top 10 MS/MS scans, and the survey of full-scan MS spectra was from 300–1750 m/z. MS scan parameters were as follows: lock mass: off, resolution: 70,000, AGC target: 3e6, and maximum IT: 50 ms. The MS/MS scans were performed at: resolution: 17,500, AGC target: 2e5, maximum IT: 100 ms, isolation window: 3.0 m/z, NCE: 25, underfill ratio: 10.0%, intensity threshold: 2.0e5, and dynamic exclusion: 30.0 s. Protein identification and label-free quantitation was performed in MaxQuant (version 1.3.2.8) using the Andromeda search engine (Cox et al., 2011). Database searching was carried out in Andromeda against the human UniProt database (October 2014 version) supplemented with contaminants. The applied parameters were: enzyme: trypsin/P; variable modifications: oxidation (M), acetyl (protein N-term), Phospho (STY), hydroxyproline and deamidation (NQ); labels: Arg10, Lys6, max. peptide PEP: 0.1; min. peptide length: 7; min. unique peptides: 1; advanced: re-quantity, keep low-scoring versions of identified peptides, match between runs (0.7 min time window), label-free quantitation, and second peptide MS2 identification. Otherwise, the default parameters of MaxQuant were used. Normalized L/H abundance ratios were calculated in MaxQuant, and the ratios of treated versus control for each cell line were analyzed for differential protein abundance using NOISeq analysis in R. Volcano plots of differential protein abundance were generated in R.

### Quantification and statistical analysis

Sigma Plot® 12.5 (Systat Software Inc.) was used for all statistical analyses with the exception of bioinformatics analyses using NOISeq, IPA and R. Single outlier detections were identified by Dixon’s and/or Grubb’s tests (threshold, P < 0.05) using ControlFreak (Contchart software). Detailed descriptions of statistical tests used, description of the number of events (n) as well as depictions of mean and standard deviations can be found in the figure legends and figures. The minimum significance level was defined as P < 0.05. The two-tailed Student’s t-test was used to test for significant differences (∗∗∗ [P < 0.001], ∗∗ [P < 0.01] and ∗ [P < 0.05]) between two samples with normally distributed parameters (Shapiro-Wilk test, P > 0.05). Mann-Whitney tests were used to test for significant differences (‡ [P < 0.01] and † [P < 0.05]) between two samples with parameters that were not normally distributed. One way ANOVA tests (Holm-Sidak method versus control) were used to test for significant differences (∗∗∗ [P < 0.001], ∗∗ [P < 0.01] and ∗ [P < 0.05]) between multiple samples with normally distributed parameters (Shapiro-Wilk test, P > 0.05). One way ANOVA on ranks tests (Dunn’s method versus control) were used to test for significant differences (‡ [P < 0.01] and † [P < 0.05]) between multiple samples with parameters that were not normally distributed. NOISeq analysis was performed in R. Sample sizes were determined based on experiment experience, pilots experiments as well as what was reported in the literature. For the NOISeq analysis, the default probability value of > 0.8 was considered significant.

## Supplemental information

Document S1. Figures S1–S7 and Table S2

Table S1. Excel file containing human tumor cell line screen results, related to Figure 1 (Sheet 1) Antiproliferation screening data for 537 human tumor cell lines upon treatment with various doses of G007-LK. After 48 and 72 h of treatment for the NCI-60 and Genentech tumor cell line panels, respectively. Tumor cell lines (477 from Genentech, highlighted by G; 24 from NCI-60, highlighted by N; 36 from both, highlighted by G + N) originating from 29 different tissues with various primary diagnoses were screened. Concentrations of G007-LK that inhibited 25% and 50% cell growth (GI _25_ and GI _50_) are shown. No determined GI _25_ or GI _50_ value is indicated by -. (Sheet 2) The 87 (16% of all) cell lines with GI _25_ values < 1 μM G007-LK listed in ascending order of GI _25_ values. (Sheet 3) Tumor cell lines (G1_25_ values < 1 μM G007-LK) sorted according to respective tissue of origin. Number and percentage of GI _25_ value hits versus the total number of tumor cell lines within tumor categories are depicted in the right columns. (Sheet 4) Tumor cell lines, with G1_50_ values < 1 μM G007-LK, sorted ascending according to respective G1_50_ values.

Table S3. Excel file containing the results of NOISeq differential expression gene/RNA sequencing analysis, related to Figure 2 (Sheets 1–5) Lists of differentially expressed genes (DEGs). The sheets contain columns depicting symbol, GeneID, log_2_ fold change, and probability. For all sheets, upon vehicle control (0.01% DMSO) and G007-LK (1 μM) treatment (24 h) of COLO 320DM, UO-31, OVCAR-4, ABC-1 and RKO tumor cell lines. (Sheets 6–9) Lists of predicted upstream regulators (UPSR). The sheets contain columns depicting upstream regulator, log expression ratio, molecule type, predicted activation state, activation Z-score, p value of overlap and target molecules in dataset. (Sheets 10–15) Lists of UPSRs selected by p value of overlap <5 × 10^−5^ and absolute activation Z-score > 0.5 or < −0.5.

Table S4. Excel file containing the results of NOISeq differential expression analysis for SILAC proteomics data for each of the cell lines and Venn analysis results, related to Figures 2 and S2 (Sheets 1–5) Lists of differentially expressed proteins. The sheets contain columns depicting symbol, control mean, treatment mean, log_2_ fold change, and probability. For Sheets 1–7, upon vehicle control (0.01% DMSO) and G007-LK (1 μM) treatment (24 h) of COLO 320DM, UO-31, OVCAR-4, ABC-1 and RKO tumor cell lines. Missing values are replaced by the value 0.3. (Sheets 6–7) Venn analysis across all cell lines for downregulated and upregulated proteins (proteins with probability >0.8 included). The sheets contain columns labeled with cell lines, total number of proteins identified, and protein name.
