# Supplementary material for: Identification of response signatures for tankyrase inhibitor treatment in tumor cell lines
Source: iScience. 2021 Jul 1;24(7):102807. doi: 10.1016/j.isci.2021.102807 (PMC8313754; doi:10.1016/j.isci.2021.102807)
Supplement: Document S1. Figures S1–S7 and Table S2 [file mmc1.pdf]

iScience, Volume *24*

Supplemental information

Identification of response signatures for tankyrase inhibitor treatment in tumor cell lines

Line Mygland, Shoshy Alam Brinch, Martin Frank Strand, Petter Angell Olsen, Aleksandra Aizenshtadt, Kaja Lund, Nina Therese Solberg, Max Lycke, Tor Espen Thorvaldsen, Sandra Espada, Dorna Misaghian, Christian M. Page, Oleg Agafonov, Ståle Nygård, Nai-Wen Chi, Eva Lin, Jenille Tan, Yihong Yu, Mike Costa, Stefan Krauss, and Jo Waaler

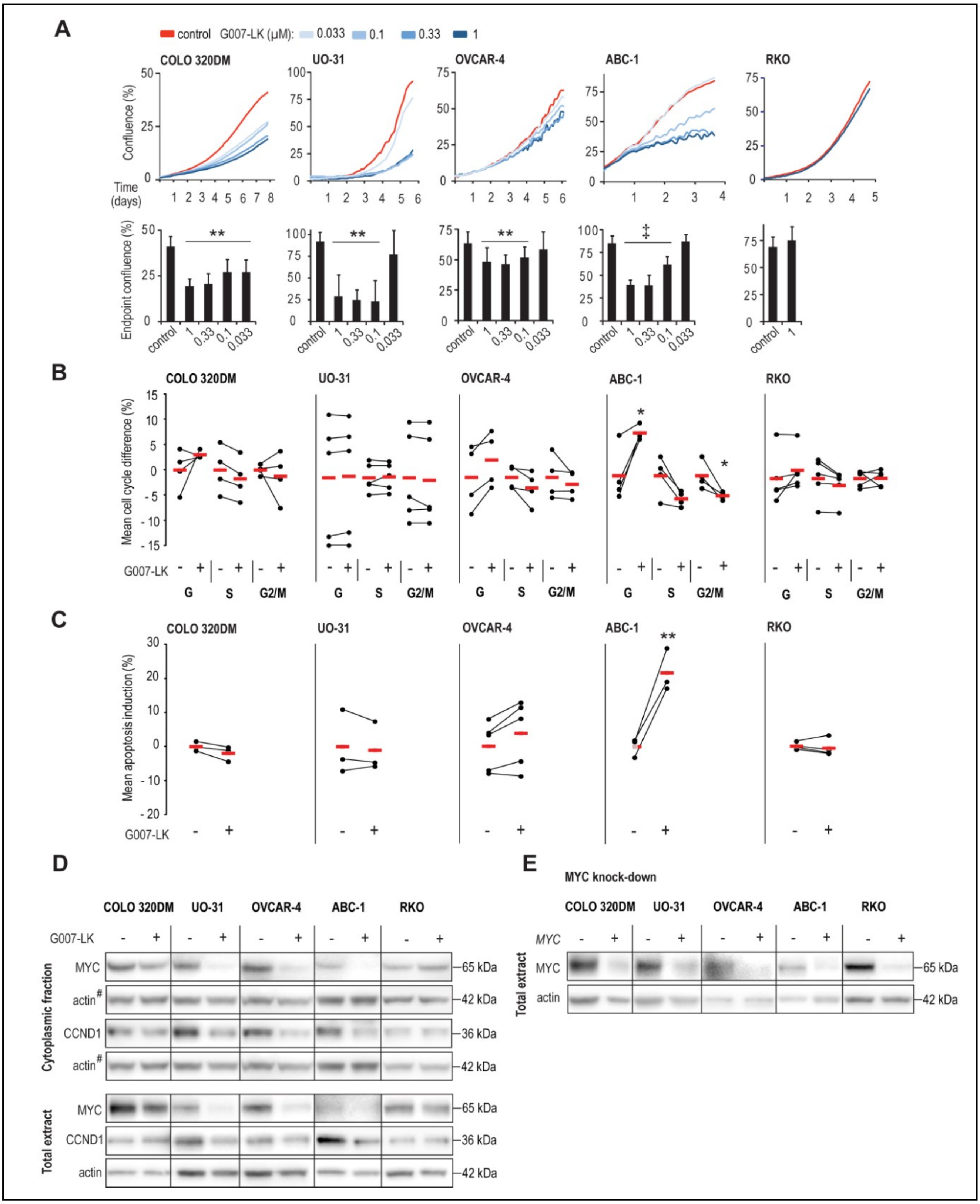
A
control G007-LK (µM): 0.033 0.1 0.33 1
COLO 320DM
UO-31
OVCAR-4
ABC-1
RKO
Confluence (%)
Time (days)
Endpoint confluence (%)
control
1
0.33
0.1
0.033
**
‡
B
Mean cell cycle difference (%)
G007-LK
G
S
G2/M
*
C
Mean apoptosis induction (%)
**
D
COLO 320DM
UO-31
OVCAR-4
ABC-1
RKO
G007-LK
MYC
actin#
CCND1
Cytoplasmic fraction
Total extract
actin
65 kDa
42 kDa
36 kDa
E
MYC knock-down
MYC
Total extract
actin
65 kDa
42 kDa

Figure S1. Proliferation screen identifies human tumor cell lines susceptible to growth inhibition by the selective tankyrase inhibitor G007-LK (related to Figure 1). (**A**) Re-screening and real-time proliferation assay (confluence, %) of the indicated concentrations of G007-LK and control (0.01% DMSO) for 4-8 days (upper panel) and the respective endpoint confluence (mean ± SD values, lower panel). For endpoint MTS data, see Figure 1C. One way ANOVA tests (Holm-Sidak method versus control) are indicated by ** ($P < 0.01$) and one way ANOVA on ranks tests (Dunn's method versus control) are indicated by ‡ ($P < 0.01$). Data from one experiment representative of at least two repeated assays with six replicates are shown. (**B**) Relative cell cycle alteration (%). G = gap_1 phase, S = synthesis phase, G2/M = gap_2/mitosis phase. Mean values (red lines) from combined data consisting of a minimum of four independent experiments are shown. For **B** and **C**, upon 72 hours treatment with G007-LK (1 μM) compared to control (0%, 0.01% DMSO). Two-tailed t-tests are indicated by ** ($P < 0.01$) and * ($P < 0.05$). (**C**) Relative induction of apoptosis (%). Mean values (red bars) from combined data consisting of a minimum of three independent experiments are shown. (**D**) Immunoblots of cytoplasmic and total extract MYC and CCND1, after 24 hours of treatment with G007-LK (1 μM) or controls (0.01% DMSO). Actin documenting protein loading and # indicates that the same actin immunblots are used as loading controls for both MYC and CCND1. For **D** and **E**, representative data from two or more independent experiments are shown. (**E**) Immunoblots of total extract MYC and CCND1, using actin documenting protein loading, 5 days after transfection with siRNA against *MYC* and *EGFP* (control).

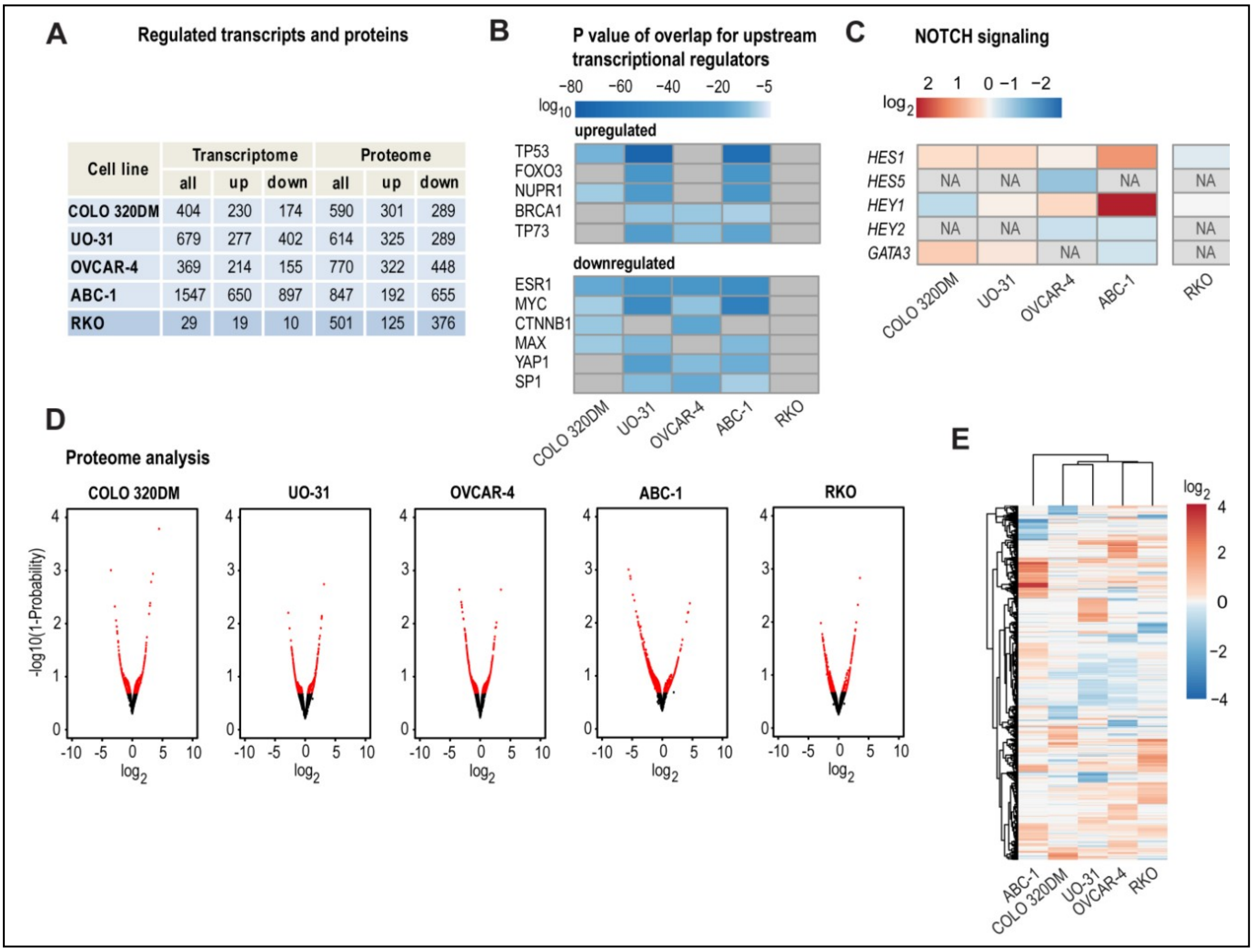

Figure S2. Gene expression and proteome analysis of TNKSi-sensitive cell lines (related to Figure 2). (**A**) Number of total (all), upregulated and downregulated genes and proteins identified upon RNA sequencing and SILAC-based proteomics analysis (probability values > 0.8).

For **A-E**, data for selected human tumor cell lines treated for 24 hours with G007-LK (1 µM, n = 2) compared to control (0.01% DMSO, n = 2). (**B**) Heatmap of P values of overlap for predicted upstream regulators (IPA core analysis) identified in ≥3 of the analyzed cell lines, plus CTNNB1 and FOXO3. Threshold: Probability values > 0.8, P value of overlap < 1×10^{-8} and absolute activation Z-score > 0.5 or < -0.5. (**C**) RNA sequencing of NOTCH signaling target gene transcripts (log_2). (**D**) Volcano plots showing the effect of G007-LK treatment on protein levels. Probability values ($-log_{10}$[1-Probability]) are plotted against log_2 fold change. Proteins with a probability values > 0.8 are highlighted in red. (**E**) Heatmap of log_2 fold change of proteins differentially expressed in at least one of the five cell lines.

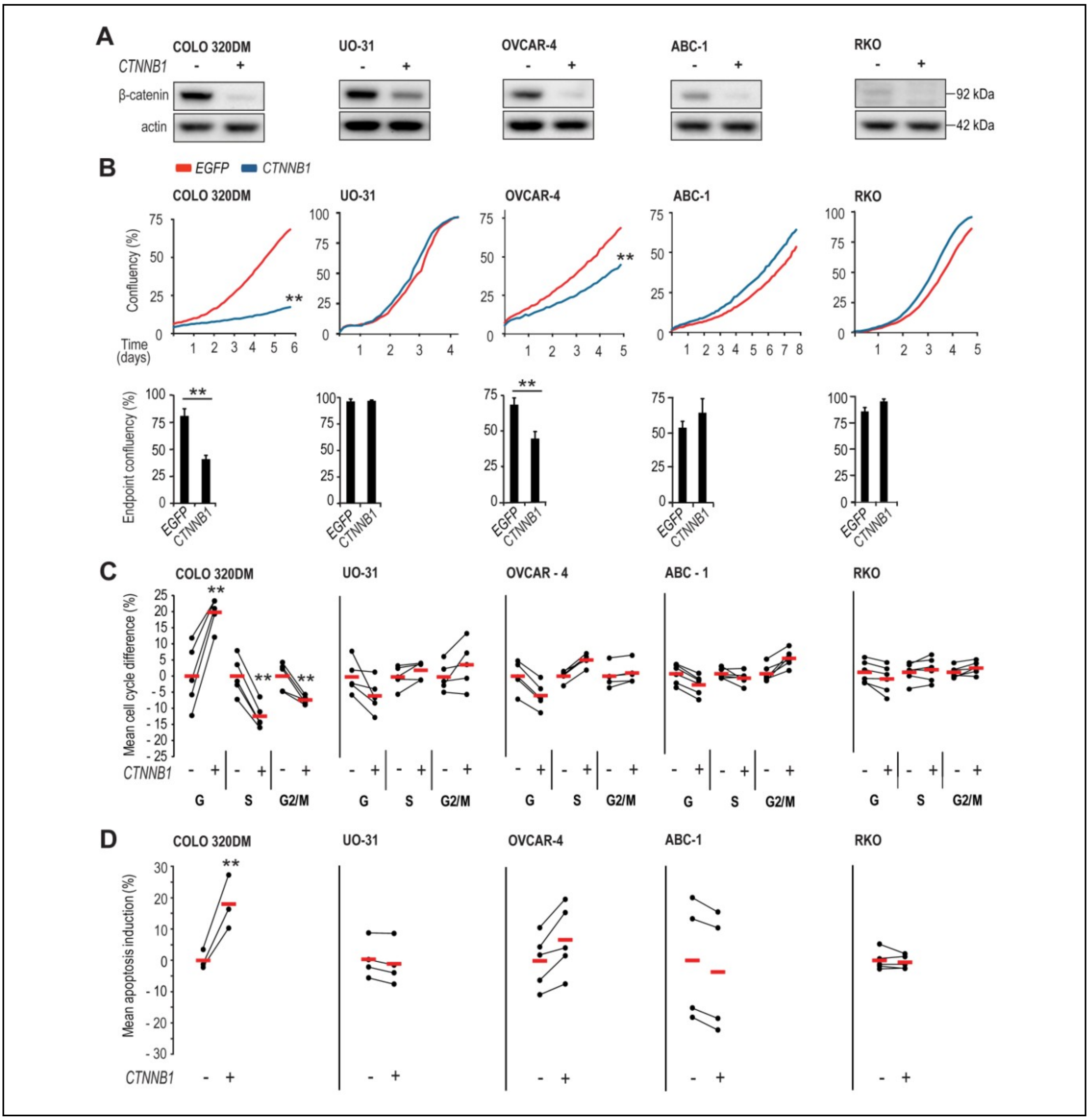

Figure S3. A subset of the tumor cell lines is dependent on β-catenin for sustained proliferation (related to Figure 3). (**A**) Immunoblots of total β-catenin upon. Actin documents protein loading. For **A**, **C** and **D**, 72 hours after transfection of siRNA against *CTNNB1* or control (0%, *EGFP*). (**B**) Real-time proliferation (% confluence) upon transfection of siRNA against *CTNNB1* (blue) or control (red, *EGFP*) for 4-8 days (upper panel) and respective endpoint confluence (mean ± SD values, lower panel). One experiment representative of at least two independent assays is shown. For **B**-**D**, two-tailed t-tests are indicated by ** ($P < 0.01$). (**C**) Relative cell cycle alteration (%). G = gap1 phase, S = synthesis phase, G2/M = gap2/mitosis phase. For **C** and **D**. Mean values (red lines) from combined data consisting of a minimum of four independent experiments are shown. (**D**) Relative induction of apoptosis (%).

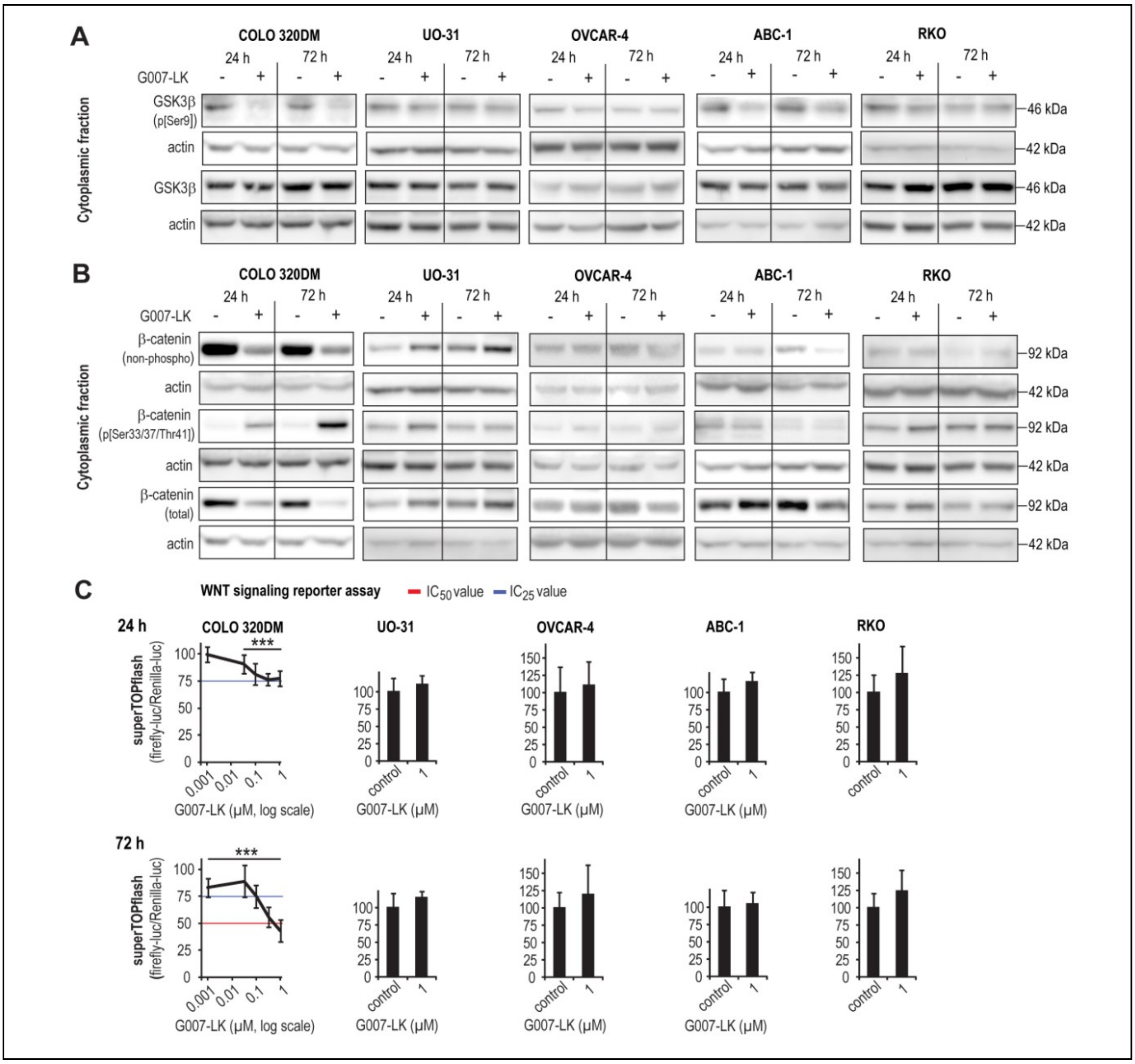

Figure S4. G007-LK inhibits WNT/β-catenin signaling in a subset of tumor cell lines (related to Figure 3 and 4). (**A**) Immunoblots of cytoplasmic fraction for inactive GSK3β (phospho[Ser9]) and total GSK3β. For **B**-**C**, after 24 or 72 hour treatment with G007-LK (1 μM) compared to controls (0.01% DMSO). Actin documents protein loading. Data representative of two or more independent experiments are shown. (**B**) Immunoblots of cytoplasmic fraction for active β-catenin (non-phospho[Ser33/37/Thr41]), inactive β-catenin (phospho[Ser33/37/Thr41]) and total β-catenin. (**C**) Luciferase-based WNT/β-catenin signaling reporter assays of cells stably transfected with SuperTOPflash and *Renilla* luciferase before treatment with different concentrations of G007-LK or control (100%, 0.01% DMSO) for 24 or 72 hours. IC_{50} (red) and IC_{25} (blue) values are depicted. One way ANOVA tests (Holm-Sidak method versus control) are indicated by *** ($P < 0.001$). Mean values ± SD for combined data from 3-5 independent experiments with three replicates each are shown.

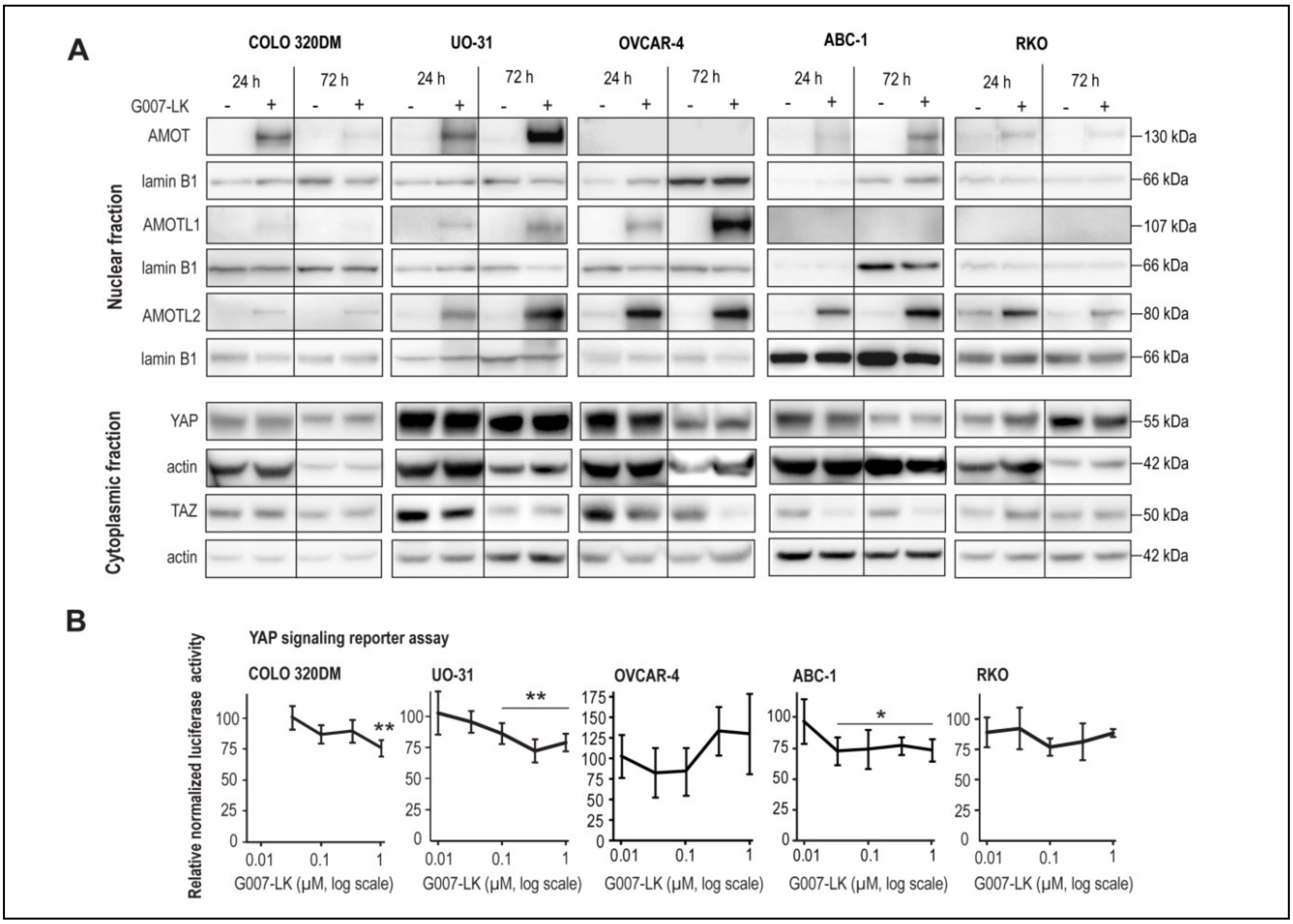

Figure S5. G007-LK stabilizes AMOT proteins and inhibits YAP signaling in the selected cell line panel (related to Figure 5 and 6). (**A**) Immunoblots of nuclear AMOT, AMOTL1 and AMOTL2 (upper panels) and cytoplasmic YAP and TAZ (lower panels) after 24 or 72 hour treatment with G007-LK (1 μM) compared to controls (0.01% DMSO). Lamin B1 and actin document protein loading. Data representative of two or more independent experiments are shown. (**B**) Luciferase-based YAP signaling reporter assays. Cells were transiently co-transfections with 8xGTIIC-driven firefly luciferase and *Renilla* luciferase (for normalization) before exposure to different concentrations of G007-LK for 24 hours compared to control (100%, 0.01% DMSO). One way ANOVA tests (Holm-Sidak method versus control) are indicated by ** ($P < 0.01$) and * ($P < 0.05$). Mean values ± SD for combined data from two independent experiments with six replicates each are shown.

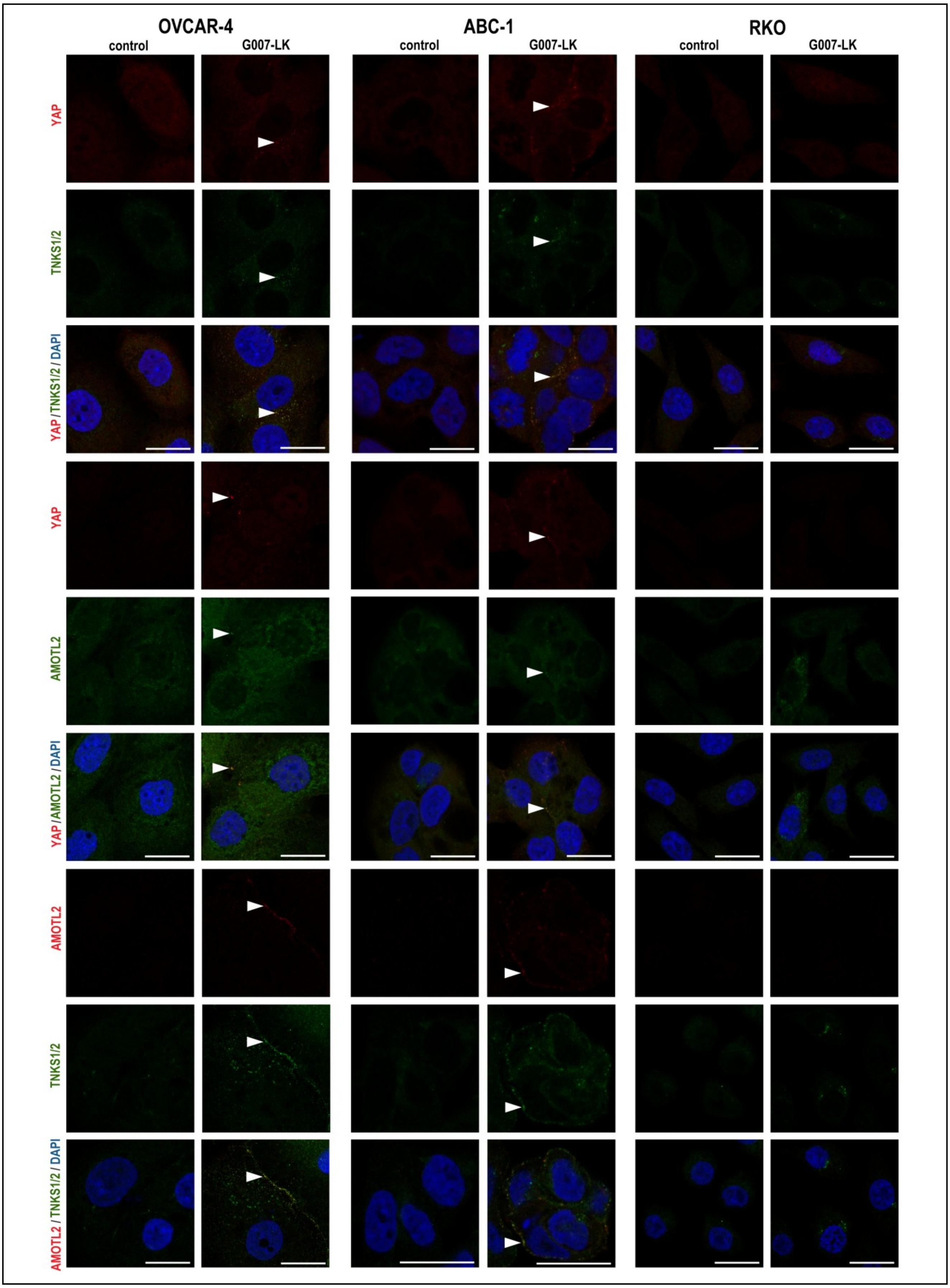
OVCAR-4
ABC-1
RKO
control
G007-LK
control
G007-LK
control
G007-LK
YAP
TNKS1/2
YAP / TNKS1/2 / DAPI
YAP
AMOTL2
YAP / AMOTL2 / DAPI
AMOTL2
TNKS1/2
AMOTL2 / TNKS1/2 / DAPI

Figure S6 (related to Figures 5 and 6). Effect of G007-LK treatment on the localization of YAP, TNKS1/2, and AMOTL2 in OVCAR-4, ABC-1 and RKO cells. Immunofluorescence staining and representative confocal images of YAP (red) and TNKS1/2 (green), YAP (red) and AMOTL2 (green) or AMOTL2 (red) and TNKS1/2 (green), along with nuclear DAPI staining (blue) upon vehicle control (0.01% DMSO) and G007-LK (1 μM) treatment (24 hours). Red, anti-mouse antibody used. Green, anti-rabbit antibody used. Arrowheads indicate co-localizations. Scale bars = 20 μm.

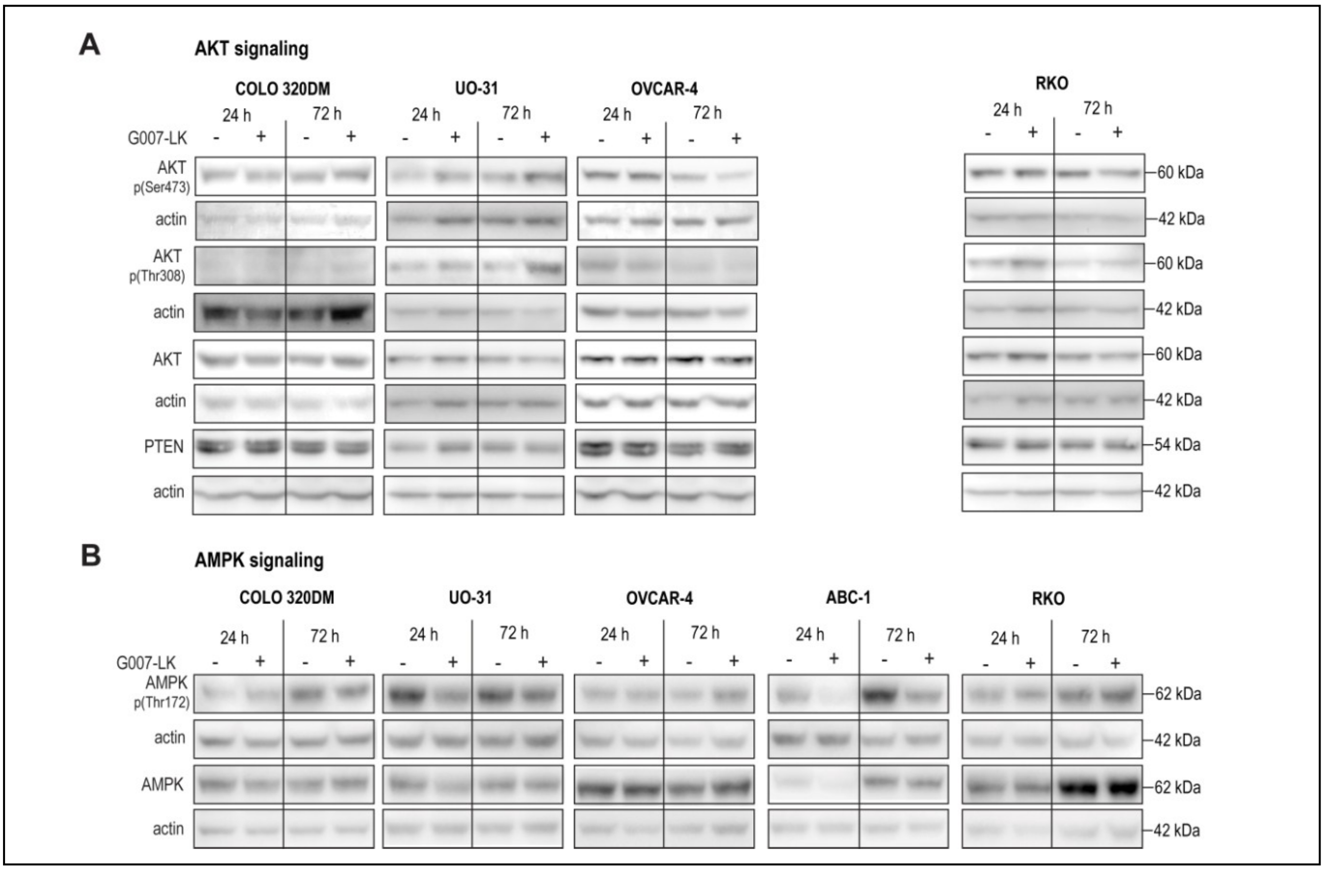

Figure S7 (related to Figure 7). Effects of G007-LK on PI3K/AKT and AMPK signaling in selected panel of human tumor cell lines. (**A**) Immunoblots of cytoplasmic fraction activate forms of AKT (phospho[Ser473] and phospho[Thr308]), total AKT and PTEN. For **A**-**B**, after 24 or 72 hour treatment with G007-LK (1 µM) compared to controls (0.01% DMSO) and actin document protein loading. Representative data from two or more independent experiments are shown. (**B**) Immunoblots of cytoplasmic active form of AMPKα (phospho[Thr172]) and total AMPKα.

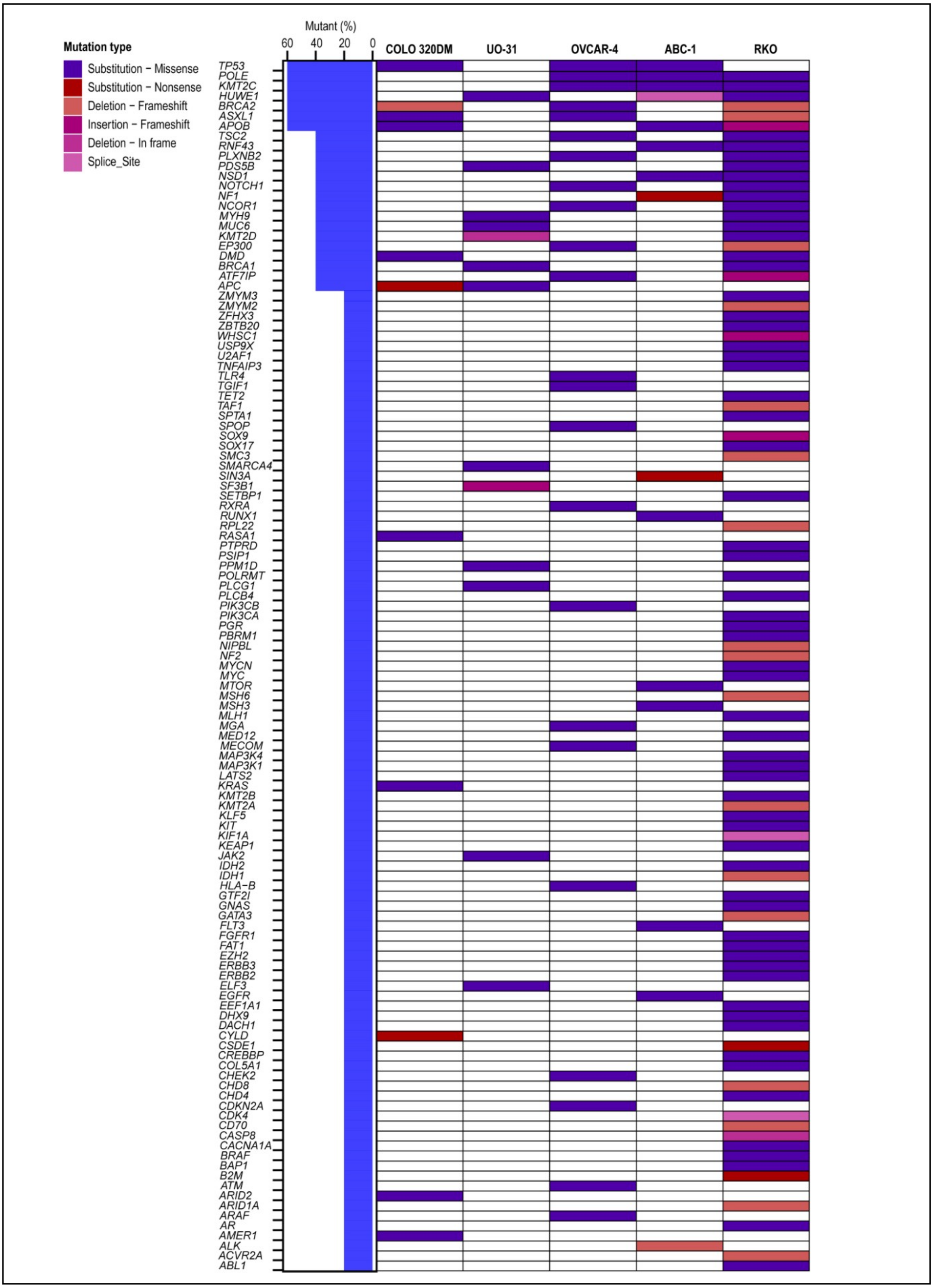

Table S2 Mutations in selected human tumor cell line panel (related to Figure 2). Waterfall plot of genomic mutations for the indicated cell lines (from CCLE, CANSAR and COSMIC databases) that match 299 previously identified driver oncogenes[5].
